# Synthesis and Characterization of Radio-Halogenated Talazoparib Analogues for Imaging and Radioligand Therapy

**DOI:** 10.1021/acschembio.6c00253

**Published:** 2026-07-27

**Authors:** Riccardo Muzzioli, Federica Pisaneschi, Seth T. Gammon, Margie N. Sutton, Arianna Napoli, Raffaele Bellini Puglielli, Lauren McIntosh, Evgeny Tereshatov, Gabriel Tabacaru, Steven Schultz, Laura McCann, Jonathan D. Burns, Sherry Yennello, David Piwnica-Worms

**Affiliations:** † Department of Cancer Systems Imaging, 4002 The University of Texas MD Anderson Cancer Center, Houston, Texas 77054, United States; ‡ Cyclotron Institute, 14736 Texas A&M University, College Station, Texas 77843, United States; § Chemistry Department, Texas A&M University, College Station, Texas 77843, United States; ∥ Department of Chemistry, 9968 The University of Alabama at Birmingham, Birmingham, Alabama 35294-1240, United States

## Abstract

Talazoparib (TZ) is a potent poly­(ADP-ribose) polymerase 1/2 (PARP1/2) inhibitor that uniquely traps PARP complexes at sites of single-strand DNA damage thereby offering opportunities for targeted radioligand therapy. Radiolabeled halogenated TZ derivatives were synthesized using boronic ester precursors to enable incorporation of diagnostic and therapeutic radionuclides: ^18^F for PET imaging, ^77^Br for Auger electron radiotherapy, and ^211^At for targeted alpha radiotherapy. Copper-mediated radio-halogenation afforded racemic ^18^F-TZ, ^77^Br-TZ, and ^211^At-TZ in sufficient radiochemical yields (4.3 ± 2.6%, *n* = 33; 29.0 ± 12.0%, *n* = 4; 3.6 ± 3.8%, *n* = 9, respectively), ∼99% radiochemical purity and proven stability under formulation conditions. Molecular dynamics simulations of halo-TZ derivatives predicted an inverse relationship between halogen size and PARP1 binding affinity. Indeed, cell uptake of radio-halogenated TZ analogues indicated selective uptake in a panel of cell types that correlated with PARP1 levels but was inversely related to the atomic radii of the halogen series. Despite modest specific activity and specific uptake, ^77^Br-TZ showed significant cytotoxicity. Further investigation of ^18^F-TZ with ^77^Br-TZ as a radiotheranostic pair will be facilitated by the synthetic schemes herein.

## Graphical abstract

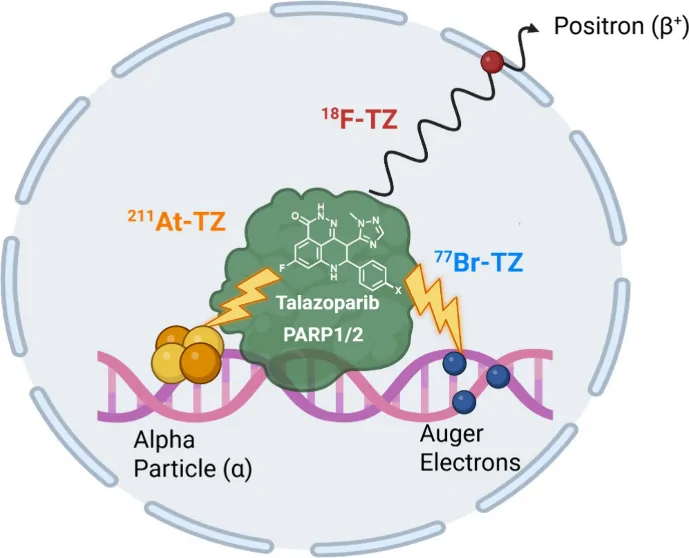

Radioisotopes are widely used in two major clinical applications: diagnostic molecular imaging (Positron Emission Tomography, PET; Single Photon Emission Computed Tomography, SPECT) and radioligand therapy (RLT) for molecular-targeted radiotherapy. RLT delivers localized radiation, i.e., beta particles, alpha particles, or Auger electrons, to tumor cells and their microenvironments while minimizing damage to healthy tissue.^1,2^ FDA-approved examples include [^131^I]­NaI, [^223^Ra]­RaCl_2_, ^177^Lu-vipivotide (Pluvicto), and ^177^Lu-dotatate (Lutathera), illustrating the proven therapeutic potential of molecularly targeted systemic radionuclides.

Among halogens, bromine, iodine, and astatine isotopes have gained attention for their combined imaging and therapeutic emission properties.^2^ Of particular interest, ^76^Br (β^+^, *t*_1/2_ = 16.2 h) supports PET imaging, while ^77^Br emits low-energy Auger electrons with subcellular ranges (10–50 Å), ideal for highly localized damage to tissue DNA or cell membranes. ^77^Br also emits gamma photons suitable for SPECT imaging.^3^ ^211^At, produced via ^209^Bi­(α,2n)^211^At, emits high linear-energy-transfer (LET) α particles and Auger electrons, followed by a longer-lived beta-emitting decay, making it attractive for blended targeted alpha radiotherapy. ^211^At also has an imageable gamma photon that is under investigation for image-based dosimetry.^4^ Together, ^18^F, ^76^Br/^77^Br, and ^211^At offer complementary imaging and radiotherapeutic capabilities, potentially forming radiotheranostic pairs within a single chemical scaffold.

Site-specific radio-halogenation of aromatic rings has been generally achieved through strategies involving the demetalation of group 14 organometallic precursors. Stannanes have been successfully used as precursors to incorporate ^18^F, ^76/77^Br, and ^211^At, and silanes and germanes are currently under investigation.^5–8^ These strategies exploit the electrophilic properties of halogens in their cationic form, which for halogens such as fluorine and, to a certain extent, bromine are difficult to stabilize. Since the pioneering work of Scott, Sanders, and Gouverneur in the early 2010s, copper-mediated deboronation–halogenation (CMBH) has become an invaluable alternative.^9–11^ Boronate precursors with good shelf stability are reliably synthesized from their corresponding aryl bromides or iodides by Suzuki–Miyaura coupling.^12^ The use of boronate precursors also mitigates the risk of nonspecific incorporation of nuclides in the electrophilic state into nonspecific sites of the target molecule, especially in the presence of aromatic rings. From the perspective of scaled production of radiopharmaceuticals, CMBH strategies employ radio-halides, which are facile to produce and stabilize. Because CMBH has been successfully used preclinically and in clinical cGMP for ^18^F-fluorination as well as for preliminary work on astatination and bromination, boronates stood out as precursors of choice when conceiving a unified strategy for the synthesis of radio-halogenated theranostics.^13–17^

^211^At has garnered increasing interest for targeted alpha therapy due to its favorable decay properties, but ^211^At chemistry remains under active development. Following production via alpha-particle bombardment of ^209^Bi, ^211^At must be separated from bismuth and other byproducts, typically through dry distillation or wet chemical methods.^18,19^ Among emerging approaches, automated solid-phase extraction using 3-octanone-impregnated Amberchrom CG300M resin has enabled isolation of ^211^At directly onto a solid support.^17^ This format facilitates safe transport and direct elution at the radiolabeling site, offering compatibility with automated synthesis modules and reducing handling complexity. Compared to traditional methods that require liquid-phase transfer or on-site distillation, solid-phase cartridges represent a practical advance in distribution logistics and workflow integration, critical for eventual incorporation into modern contract drug manufacturing processes. Most astatination strategies have mirrored classic electrophilic ipso-demetalation reactions from stannane or organomercury precursors, assuming electrophilic behavior of astatine. More recently, nucleophilic astatination of aryl boronic esters has emerged as a promising alternative, enabling incorporation of ^211^At into biologically relevant molecules, including PARP inhibitors.^16^

Poly­(ADP-ribose) polymerase 1 (PARP1) is a nuclear enzyme essential for base excision repair of single-strand DNA breaks and has recently been heavily explored as a target for radioligand therapy.^20^ PARP inhibition induces synthetic lethality and orthogonal immune responses in BRCA-mutated cancers, establishing PARP inhibitors as key therapeutics.^21–23^ Talazoparib (TZ), an FDA-approved PARP1/2 inhibitor, exhibits nanomolar potency and slow dissociation and is thought to “trap” PARP1 complexes at DNA damage sites in living cells, amplifying cytotoxicity.^24^ The long residency time of the TZ/PARP1/DNA complex makes TZ an attractive candidate for Auger and alpha radioligand therapy, as radionuclides held for sufficient time in close proximity to DNA could maximize localized damage. Indeed, the nanometer LET range for Auger electrons may provide a level of selectivity for PARP1/DNA damage complexes in tumor cells, potentially critical for translational targeting of tumors with pan-PARP-targeted radiotheranostics due to on-target off-tissue expression in a variety of tissues with heterogeneous and context-dependent subcellular localization.^25^ Current PARP imaging agents, such as ^18^F-FluorThanatrace (^18^F-FTT) and ^18^F-PARPi, are based on rucaparib or olaparib scaffolds.^26,27^ Radiolabeled analogues of these inhibitors also show *in vitro* Auger cytotoxicity and potential for systemic radioligand therapy.^28^ However, unlike the modifiable carbon sites on rucaparib or olaparib that direct substituents out of the PARP binding pocket and into the solvent, the halogens of TZ lie within the PARP binding pocket. This geometry likely contributes to the superior affinity of TZ and slow off rate, critical for potentially enhanced radiotherapy. However, this binding geometry complicates synthetic access for radiolabeling of TZ via pendant groups. A unified labeling strategy is therefore desired for efficient synthesis and evaluation of radio-fluorinated TZ along with additional radio-halogenated TZ analogs.^29^

Herein, we report a robust, unified, copper-mediated approach for radiolabeling TZ with ^18^F, ^77^Br, and ^211^At using boronic ester precursors. This synthetic scheme eliminated the need for radically different radionuclide-specific precursors and their associated workup time and effort while enabling the synthesis of ^18^F-TZ for PET and cell uptake studies, ^77^Br-TZ for Auger electron RLT, and ^211^At-TZ for combined alpha and Auger RLT. This unified strategy would enable the facile evaluation of multiple tracers on the TZ platform for rapid appraisal of the potential for imaging and RLT.

To start, inspection of the space-filling TZ crystal structure (Table S1) identified the *para*-phenyl fluorine as a suitable site for modification with radio-halogens. This position was expected to be relatively far from the interaction site in PARP and exhibit increased chemical reactivity, thereby facilitating precursor synthesis and subsequent radiolabeling. Among the actionable radio-halogens, a subset representing alternating elements across the halogen series was selected to efficiently span steric size and therapeutic potential, from imaging to Auger to alpha therapy indicated by color (fluorine red, bromine blue, astatine yellow) (Figure 1A, left). Initially, racemic [(8*S*,9*R*)/(8*R*,9*S*)] boronic ester precursors were synthesized from 6-fluoro-4-nitroisobenzofuran-1­(3*H*)-one and 1-methyl-1*H*-1,2,4-triazole-5-carbaldehyde via a modified literature procedure (Scheme S1A,B). The racemic (2-(trimethylsilyl)­ethoxy)­methyl (SEM)-protected boronic ester (**1**; Figure 1A, right) was isolated in 3.3% overall yield, while the unprotected analog (**2**; Figure 1A, right) was obtained in 27% overall yield. Precursors were stored in a desiccator at 4 °C in the dark, which was sufficient to enable labeling for several months (Supporting Information). Similarly, racemic bromo-talazoparib (Br-TZ) (**7**, Scheme S1A) and racemic iodo-talazoparib (I-TZ) (**11**, Scheme S1C) were obtained in 63% and 54% overall yield, respectively. All compounds were fully characterized by ^1^H/^13^C NMR and MS (Supporting Information).

**Figure 1:**
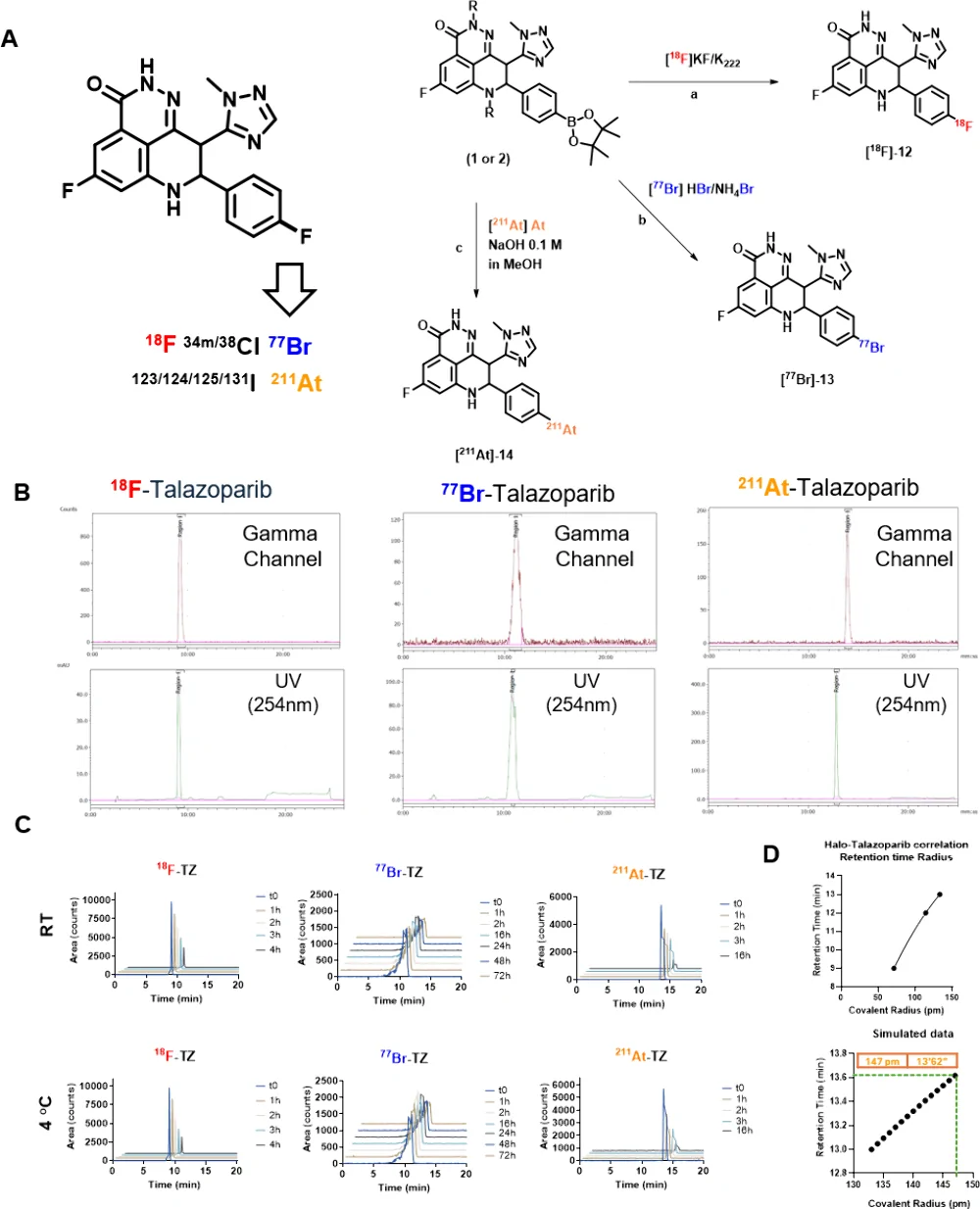
Radiosynthesis and characterization of radio-halo-talazoparib. (A) Structures of TZ derivatives (left). Alternating elements across the halogen series were selected to efficiently span steric size and therapeutic potential, from imaging to Auger to alpha therapy indicated by color (fluorine red, bromine blue, astatine yellow). Strategy for synthesis is shown (right); R = SEM group (**1**) or H (**2**). Condition a: [Cu­(OTf)_2_(Py)_4_], DMF, 120 °C and 4 M HCl for deprotection. Condition b: [Cu­(OTf)_2_(Py)_4_], DMSO, 120 °C and 4 M HCl for deprotection. Condition c: [Cu­(OTf)_2_(Py)_4_], 3,4,7,8-tetramethyl-1,10-phenanthroline, RT. (B) Radio-purity and identity assessed by HPLC. I-TZ was used as a surrogate for At-TZ. (C) Assessment of radio-TZ shelf stability in PBS/10% EtOH at rt and 4 °C at different time points. (D) Correlation between retention time of halogenated TZ and atomic radius (pm) of the halogen bound to the TZ scaffold (under the same HPLC conditions); nonlinear regression (GraphPad) was used to simulate retention time for astatine. All parameters exceeded requirements for preclinical evaluation and were potentially scalable for downstream biomedical applications.

^18^F-TZ, racemic, was synthesized on a GE TracerLab FX automated module via copper-mediated deboronation-radiofluorination of the SEM-protected boronic ester (**1**; Figure 1A), affording ^18^F-TZ in 4.3 ± 2.6% yield (*n* = 33) and >99% radiochemical purity.^30^ Identity was confirmed by co-injection with TZ (Figure 1B). ^77^Br-TZ, racemic, or ^211^At-TZ, racemic, were similarly synthesized from the SEM-protected precursor (**1**; Figure 1A) or from the nonprotected precursor (**2**; Figure 1A), respectively, using a manual synthetic procedure amenable to transfer to an automated synthesizer. Identity was confirmed by radio-HPLC using Br-TZ or I-TZ as a surrogate in the case of ^211^At-TZ (Figure 1B). Radio-halogenated TZ derivatives were highly stable over time consistent with isotope half-lives (Figure 1C). Interestingly, HPLC retention times of the halogenated F-, Br-, and I-TZ series correlated with their covalent radii (pm); the retention time of ^211^At-TZ was predicted by nonlinear regression (Figure 1D). The predicted retention time of 13.6 min for the 147 pm radius of ^211^At corresponded well with the experimental value of 13.5 min. Yields, molar activity, and LogP/LogD analysis are summarized in Table 1. LogP/LogD values were consistent with predicted values (range 1.1–2.0, Molinspiration property engine).

**Table 1:** Summary of Synthesis and Radiochemical Analysis

Compound	Radiochemical Yield	Molar Activity (GBq/μmol)	Log P/Log D
^18^F-Talazoparib	4.3 ± 2.6% (SD, *n* = 33)	3.0	1.9/1.9
^77^Br-Talazoparib	29.0 ± 12.0% (SD, *n* = 4)	0.1	1.7/1.7
^211^At-Talazoparib	3.6 ± 3.8% (SD, *n* = 9)	1.4	1.6/1.6

*In silico* binding to PARP1 of enantiopure TZ and derivatives bearing chlorine, bromine, iodine, and astatine substitutions (X) were determined (Autodock Vina 1.2.0, PDB = 7kk3, CDBio) (Figure 2). For validation, excellent concordance was observed between the *in silico* model of TZ bound to PARP1 and the published crystal structure coordinates of TZ bound to human PARP1 ^31^ (Figure 2B). Calculated binding energies were −10.83 kcal/mol (TZ), −9.11 kcal/mol (Br-TZ), −8.98 kcal/mol (I-TZ), and −8.68 kcal/mol (At-TZ), revealing an inverse correlation between halogen covalent radii and PARP1 binding affinity (Figure 2C,D). Molecular modeling revealed a positional docking shift as a function of halogen radii. Halogens bulkier than fluorine reoriented the molecule within the pocket, with secondary local affinity adjustments occurring monotonically from bromine to astatine (Figure 2C). For radiotherapeutic halogens, there is a reduced strength of interaction with an expected ∼7-fold (Br-TZ) and 27-fold (I-TZ) change in predicted affinity from fluorine. These data were not used to generate actual *K*_d_ values but rather to rank order and prioritize halogens for potential correlation with relative cell uptake values. Note that cell-free PARP1 enzyme activity assays showed IC_50_ inhibition values for nonradioactive racemic Br-TZ and I-TZ of 2.3 nM and 100 nM, respectively (Figure S1), consistent with the rank order of calculated binding energies (Figure 2D). Furthermore, preliminary cytotoxicity assays showed sub-micromolar activity for cold racemic I-TZ consistent with a modest IC_50_ *in vitro* (Figure S2).

**Figure 2:**
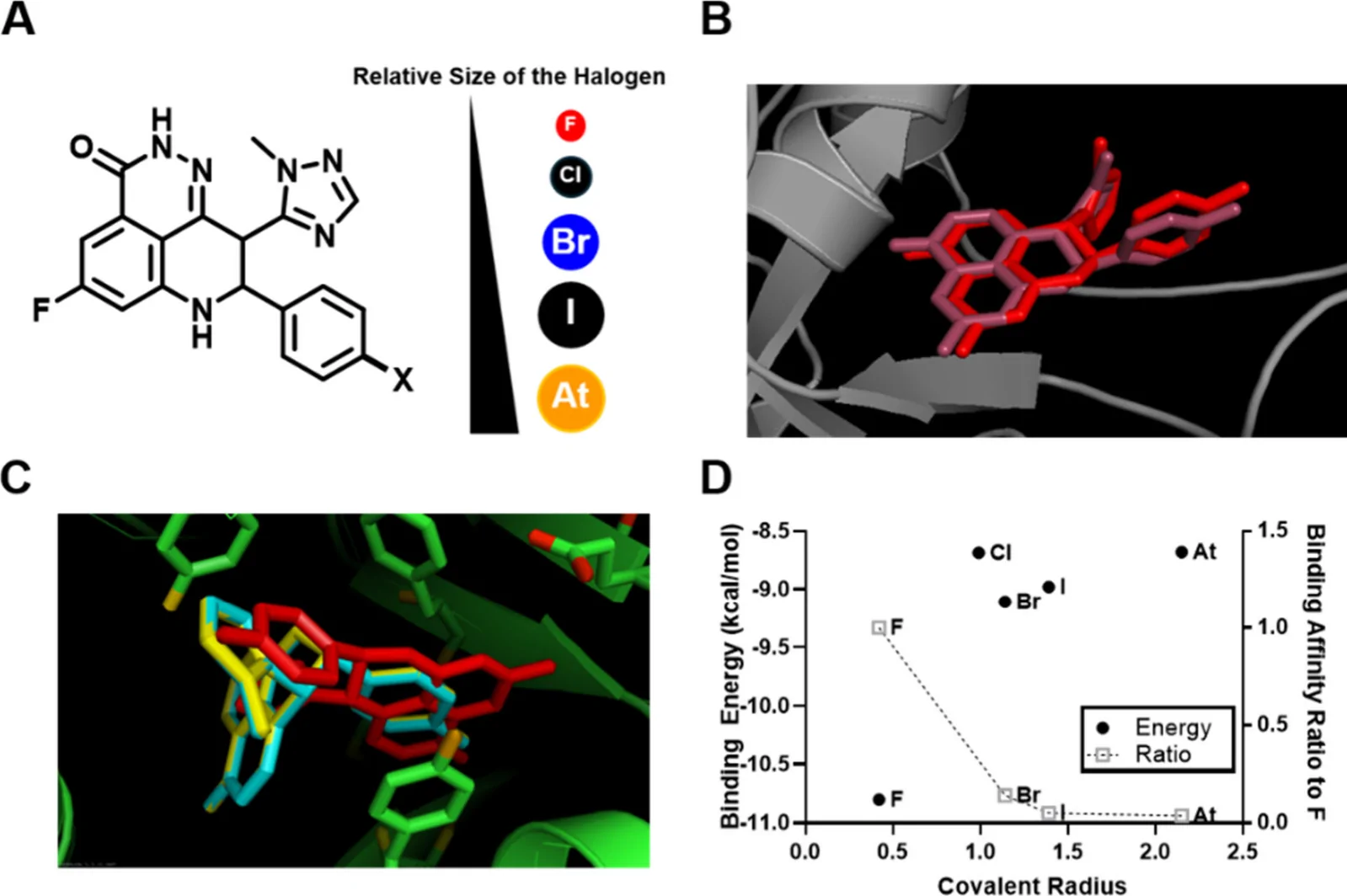
Molecular modeling of talazoparib analogues. (A) Structure of TZ. Aryl halogens (X) are shown as scaled relative atomic radii. (B) Molecular modeling of TZ docked within the PARP1 binding site (red) compared to the crystal structure of bound TZ (purple). (C) Molecular modeling of enantiopure TZ (red, protein green), Br-TZ (blue), and At-TZ (yellow) docked in PARP1. (D) Docking of TZ analogues yielded dependence of binding energy on the covalent atomic radius of X (solid symbol) with a projected decline in affinity relative to TZ for the radiotherapeutic halogens. A dominant potential loss in affinity appears to be reorientation of TZ analogues in the binding pocket for halogens larger than fluorine.

Radioactive racemic halo-TZ cell uptake was evaluated in cell lines with varying PARP1 protein levels and BRCA1 status to enable preliminary evaluation of predictive biomarkers of cell net uptake and cytotoxicity (Figure 3). Given radiosynthesis-driven constraints in molar activities, total extracellular concentrations (EC) of tested TZ derivatives were similar but not identical. For racemic ^18^F-TZ (EC 10 nM), cell uptake at 37 °C massively exceeded that at 4 °C (Figure 3A). Pretreatment with excess nonradioactive TZ completely blocked ^18^F-TZ uptake, confirming high specificity binding to TZ targets (i.e., PARP1/2 and perhaps unspecified membrane transporters) relative to expectations for high-capacity nonsaturable binding, such as membrane partitioning. ^18^F-TZ uptake correlated with normalized PARP1 protein (Spearman *r* = 0.7; *p* = 0.11 one-tailed), *PARP2/3* mRNA, and total *PARP* mRNA (Figure 3D,E,F). Under similar conditions, ^77^Br-TZ (EC 40 nM), demonstrated consistent but modest cell uptake above background and 4 °C (Figure 3B). Given the cold-sensitivity of metabolically or membrane-transport-detectable cell uptake as well as modeling evidence of preserved points of contact in the binding pocket (Figure 3G), ^77^Br-TZ was further tested for cytotoxicity in a 72 h SRB cell viability assay against the cell line panel in experiments performed at the expected maximum human blood concentration (Figure 3H; for dosimetry estimates see Supporting Information). Significant cytotoxicity was detectable versus key controls for all cell lines tested (2-way ANOVA, corrected for multiple testing). As suggested from the low specific cell uptake data, ^77^Br-TZ-treated cell viability negatively correlated with *PARP1* mRNA levels but, interestingly, correlated modestly with *PARP2* and *PARP3* mRNA levels and *BRCA1* status of the various cell lines (Figure 3I). At the available molar activity, cell uptake of ^211^At-TZ (EC 20 nM) could not be reliably detected above nonspecific binding to plastic substrate, 4 °C controls, or assay noise (Figure 3C), and thus, cytotoxicity evaluation could not be performed.

**Figure 3:**
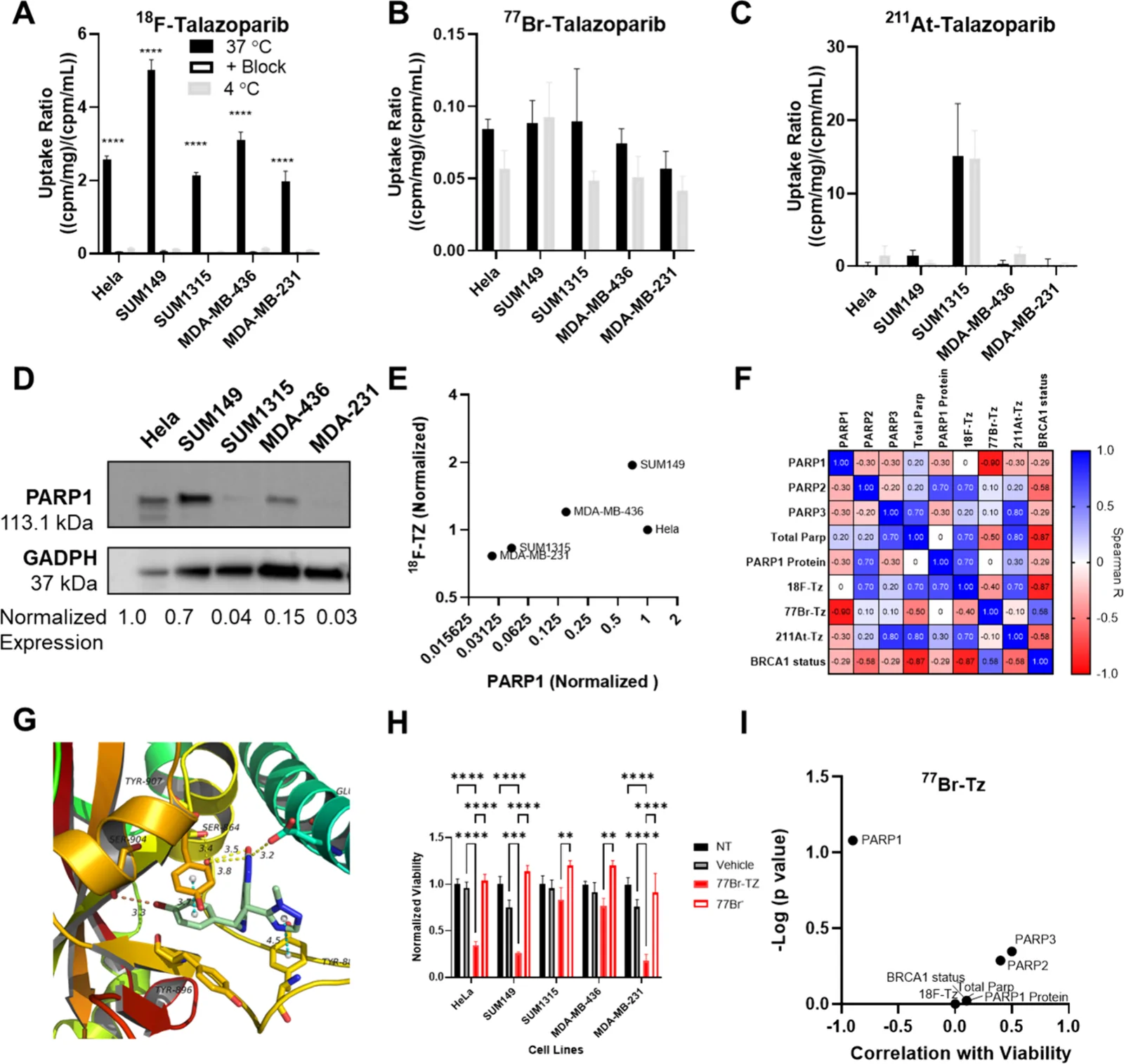
Cell uptake, cytotoxicity, and potential biomarkers for halo-talazoparib efficacy. Cell uptake was determined for racemic ^18^F-TZ (A), ^77^Br-TZ (B), and ^211^At-TZ (C) across a variety of cell lines, including two BRCA± breast cancer cell line pairs (SUM149/SUM1315; MDA-436/MDA-231) and HeLa cells serving as a PARP1-expressing, non-breast-cancer cell line. (D) PARP1 protein levels were assessed by immunoblotting and normalized to GAPDH and HeLa cell PARP1. (E) Cell uptake of ^18^F-TZ correlated with normalized PARP1 (*r* = 0.7, one-tailed *p* = 0.11). (F) Wider spectrum of potential theranostic co-correlates was also evaluated. (G) Modeling of Br-TZ binding to PARP1 revealed the preservation of several key contacts, suggesting the possibility of modest binding. (H) Cell viability was determined after 72 h of treatment with ^77^Br^(−)^ or ^77^Br-TZ at 0.42 MBq/mL (0.011 mCi/mL). Test for significance was a 2-way ANOVA with multiplicity correction for post hoc comparison of ^77^Br-TZ versus all three controls (untreated, vehicle/ethanol treated, and ^77^Br^(−)^). (I) Viability was correlated with various parameters potentially related to ^77^Br-TZ cytotoxicity, including BRCA1 status binarily defined as positive or negative BRCA activity based upon the literature. ****p < 0.0001; **p < 0.01.

While potent nonradioactive PARP inhibitors contribute to clinical practice, resistance mechanisms limit their long-term efficacy.^32^ Escape routes can include downregulation of PARP, overexpression of multidrug resistance transporters (e.g., MDR1), upregulation of homologous recombination repair, or reversion mutations in *BRCA1/2*.^32^ Thus, within the domain of biomedical applications, escape mutants that preserve the PARP target, such as HR recombination or BRCA repair, create a niche opportunity for radioligand therapy, while PET diagnostics may integrate information regarding target loss via either PARP downregulation or upregulation of efflux transporters.^33–37^

The unified copper-mediated strategy for radiolabeling TZ with ^18^F, ^77^Br, and ^211^At from a single boronic ester precursor provides a practical framework for exploring this concept. By enabling rapid synthesis and evaluation of multiple isotopes under biologically relevant conditions, this approach may accelerate identification of candidates that combine imaging and radiotherapeutic potential compared to timelines that require novel precursors with novel radiolabeling strategies for each test item. In addition, the retention time correlation study proved the utility of iodo-derivatives as standards to confirm the identity of ^211^At-labeled compounds, important as there is no nonradioactive isotope of astatine that can be produced at mass scale. For the present study, the recently reported automated method for on-resin ^211^At delivery was exploited, facilitating shipment and manipulation of this short-lived alpha-emitting radio-halogen.^17^

Herein, with a focus on ^77^Br-TZ, significant cytotoxicity at clinically relevant radioactive concentrations was observed, underscoring the potency of Auger electrons when delivered in proximity to DNA. Note that cell uptake and cytotoxicity results for ^77^Br-TZ and ^211^At-TZ were challenged by the modest molar activities available for the studies (0.1 and 1.4 GBq/μmol, respectively). These data highlighted the potential for Auger emitters on this scaffold if molar activity is further optimized in the development process. Finally, while cell uptake of ^18^F-TZ correlated with PARP1 protein levels as found with ^18^F-FTT,^26^ cell uptake of ^77^Br-TZ did not correlate with PARP1 protein levels at the molar activities tested. However, interestingly, viability of cells when treated with ^77^Br-TZ trended to negatively correlate (but NS) with *PARP1* mRNA levels as expected, suggesting that indeed Auger targeting might bypass key resistance mechanisms of PARPi treatment, including rescue of functional BRCA pathways. Alternatively, there could be saturation or halogen mismatch with a yet undetermined inward membrane transport system given that there are known and reversible efflux transporters for PARP inhibitors broadly and talazoparib specifically that might confound simple linear correlations of halogen size and cell uptake or efficacy.^37,38^ These findings highlight the importance of iterative integration of radiosynthesis, modeling, and biological assays to prioritize isotopes for scenarios where conventional PARP inhibition fails.

Note that the radio-halogen TZ derivatives herein were racemic mixtures, and comparison to recent data by others provided opportunities to inform mechanisms of PARPi pharmacology. For example, the racemic cold I-TZ IC_50_ of cell-free PARP1 enzyme activity was 100-fold higher than the IC_50_ of enantiomerically pure TZ (0.6 nM). While use of different commercial kits may influence determination of IC_50_ values due to different concentrations of competitive cofactors and substrates, the more interesting hypothesis is that for the larger halogen-TZ enantiomers, the “inactive” enantiomers may not in fact be inert but rather interfere pharmacologically (noncompetitive, uncompetitive) with substrate binding in the PARP active site. Indeed, enantiomeric interference may apply to both PARP enzymes as well as putative membrane transporters. This model is consistent with recent live cell radio-I-TZ data whereby a racemic mixture of ^123^I-TZ had far less than half the uptake of the active enantiomer minus the inactive enantiomer, implying interference.^39^

Furthermore, the surprising success of ^77^Br Auger emissions even at modest molar activities primarily represents a potential false negative risk for assessment of other therapeutics, including beta emitters. Specifically, absolute cytotoxicity and cell uptake assays may improve at higher molar activity and enantiopurity while preserving the halogen rank order to enhance the potential for Auger therapy. Indeed, Auger electron dosimetry can nonlinearly correlate with clonogenic efficacy^40^ yet underestimate both toxicity and immunogenicity as also reviewed by Kassis et al.^1,41^ Thus, testing of other Auger emitters, including ^123/125^I, while balancing the dosimetry risk of partial dehalogenation for large unsubstituted aryl halogens^8,42^ against the emerging supply chain for ^77^Br and ^211^At should continue to be considered.

Rather than competing with existing therapies, this work establishes a potential route for precision intervention in mechanistic escape mutants, wherein additional PARP protein family members remain present, accessible, and trappable. The ability to adapt a precursor strategy for multiple isotopes is critical to enabling rapid design iterations, offering a blueprint for advancing small-molecule radiotheranostic strategies in oncology, particularly for established direct injection therapies where bone marrow and liver trapping are bypassed and long residency and high potency are favored.

## Materials and Methods

### Chemicals and Reagents

All reagents were obtained from commercial sources and used without any further purification. Anhydrous solvents were purchased from Thermo Fisher Scientific. Radioisotopes ^18^F, ^77^Br, and ^211^At were supplied by institutional cyclotron facilities: UT MD Anderson Cancer Center, Washington University in St. Louis, or University of Wisconsin, and Texas A&M, respectively. Radioactivity was measured using a Capintec CRC-15R dose calibrator. PCR kits were obtained from Bio-Rad. Sequences for forward and reverse primer sequences were obtained from Primerbank with primerbank ID 156523967c1, 110825962c1, and 158634481c2 for *PARP1*, *PARP2*, and *PARP3*; primers were ordered from Sigma.^43^ An anti-PARP1 antibody (46D11 rabbit monoclonal, Cell Signaling) was utilized for immunoblot characterization of protein levels.

### Chemical Synthesis

Racemic talazoparib analogs (Br-TZ, I-TZ) and boronic ester precursors were synthesized from 6-fluoro-4-nitroisobenzofuran-1­(3*H*)-one and 1-methyl-1*H*-1,2,4-triazole-5-carbaldehyde via modified literature procedures using key intermediates identified from the published synthesis for talazoparib.^8,44–46^ SEM-protected and unprotected boronic esters were prepared for radiolabeling.^47^ Structures were confirmed by ^1^H/^13^C NMR and MS (ESI).

### Analytical Methods

QC was performed by analytical radio-HPLC with UV and radiodetection. NMR spectra were recorded on Bruker 500/600 MHz instruments; MS analysis used a Xevo TQD with ESI.

### Radiosynthesis

Radiolabeling was performed via copper-mediated halogenation of boronic ester precursors. ^18^F-TZ was synthesized on a GE TracerLab FX using [Cu­(OTf)_2_(Py)_4_] in DMF at 120 °C, followed by acid deprotection and HPLC purification. ^77^Br-TZ was manually synthesized in DMSO at 120 °C with [Cu­(OTf)_2_(Py)_4_], followed by acid deprotection and HPLC purification. ^211^At-TZ was manually synthesized using unprotected boronic ester in ACN/EtOH with [Cu­(OTf)_2_(Py)_4_] and 3,4,7,8-tetramethyl-1,10-phenanthroline, purified by HPLC. All radiolabeled compounds were reformulated in PBS/10% ethanol and confirmed by co-injection with nonradioactive standards (note I-TZ for ^211^At-TZ). Radiochemical purity exceeded 99%.

### Stability

Formulated radiolabeled compounds were stored at 4 °C or RT in PBS/10% ethanol. Stability was assessed by radio-HPLC at defined intervals (Figure 1) based on isotope half-life: 0–4 h, ^18^F; 0–72 h, ^77^Br; 0–16 h, ^211^At.

### Cell Culture, Radiotracer Uptake Studies, and Cytotoxicity Assay

Human cancer cell lines (HeLa, SUM149, SUM1315, MDA-MB-231, MDA-MB-436) were obtained from registered sources and cultured under standard conditions with appropriate media. Cell radiotracer uptake assays were performed by incubating cells with radiolabeled halo-TZ analogues (∼1 μCi/mL (0.037 MBq/mL) or up to 2 μL/mL vehicle) for 120 min at 37 or 4 °C, followed by a brief wash (4 °C) to clear extracellular tracer. Blocking studies used molar excess nonradioactive talazoparib. Cell-associated radioactivity was quantified by gamma counting and normalized to cell protein content (BCA assay) and extracellular tracer concentration, expressed herein as (cpm/mg protein)/(cpm/mL). Published estimates for mg protein per cell^48^ and volume per cell for HeLa cells^49^ and others enabled cell dosimetry calculations (Supporting Information).

## Supplementary Material



## Notes

Additional experimental details, materials, and methods, ^1^H/^13^C NMR spectra and MS for all compounds, TZ binding interactions, compound stability, molar activity calculations, dose estimates, qPCR *PARP* primers, cell-free PARP1 inhibition data, and cell cytotoxicity data for racemic I-TZ (PDF)

## ◇

Solve Therapeutics, Durham, North Carolina 27713, United States

## #

UT Health Sciences Center of Houston, Center for Translational Cancer Research, The Brown Foundation Institute for Molecular Medicine, 7000 Fannin St., Houston, Texas 77030, United States.

## &

Nusano Inc., West Valley City, UT, 84120, United States.

## Abbreviations

NMR: nuclear magnetic resonance
MS: mass spectrometry
