# Supplementary material for: Synthesis and Characterization of Radio-Halogenated Talazoparib Analogues for Imaging and Radioligand Therapy
Source: ACS Chem Biol. 2026 Jul 27;21(8):1883–90. doi: 10.1021/acschembio.6c00253 (PMC13505185; doi:10.1021/acschembio.6c00253)
Supplement: Supplementary file 1 [file cb6c00253_si_001.pdf]

Supporting Information

Synthesis and Characterization of Radio-Halogenated Talazoparib Analogues for Imaging and Radioligand Therapy

Riccardo Muzzioli[1†], Federica Pisaneschi[1††], Seth T. Gammon[1], Margie N. Sutton[1], Arianna Napoli[1], Raffaele Bellini Puglielli[1], Lauren McIntosh[2], Evgeny Tereshatov[2], Gabriel Tabacaru[2], Steven Schultz[2,3], Laura McCann[2,3], Jonathan D. Burns[4], Sherry Yennello[2,3], David Piwnica-Worms[1]*

[1] Department of Cancer Systems Imaging, The University of Texas MD Anderson Cancer Center, Houston, TX, USA

[2] Cyclotron Institute, Texas A&M University, College Station, TX, USA

[3] Chemistry Department, Texas A&M University, College Station, TX, USA

[4] Department of Chemistry, The University of Alabama at Birmingham, Birmingham, AL, USA

Supporting Information

Extended Methods and Compound Characterization.

All chemicals were purchased from ThermoFisher Scientific (Waltham, MA), Enamine, Selleckchem, Sigma-Aldrich (St. Louis, MO) and used without further purification. Anhydrous solvents were purchased from Thermo-Fisher Scientific.

Reactions were monitored by UPLC-MS system carried out on Waters Acquiy UPLC I class plus using an Acquity UPLC C18 1.7µm (2.1x50 mm) column under the following condition: Solvent A: H_2O + 0.1% formic acid; Solvent B: Acetonitrile (ACN) + 0.1% formic acid; Gradient: three minutes (10%-95% B). The samples from the column were injected in a Xevo TQD (Z spray ESI, APCI) mass spectrometer.

Preparative flash chromatography was performed using prepacked silica cartridge (Silicycle 25µm Premium) 40g, 80g, 100g on an automated chromatography system (Biotage Isolera1) using dichloromethane (DCM) and methanol (MeOH) as solvent system and presented a UV detector and an automatic fraction collector. All NMR spectra were recorded on Bruker ultra-shield 500MHz or Ultra Shiled 600MHz plus spectrometer. Proton (1H) and carbon-13 NMR (13C) spectra are reported as chemical shifts (δ) in parts per million (ppm) relative to the solvent residues peak as reference or Me_4Si as internal standard. Coupling constants (J) are reported in units of hertz (Hz). The following abbreviations are used to describe multiplicities – s (singlet), d (doublet), t (triplet), q (quartet), m (multiplet), brs (broad singlet). Compounds were dissolved in deuterated chloroform ($CDCl_3$, solvent residues δH: 7.26ppm, δC: 77.16 ppm) or deuterated dimethyl sulfoxide (DMSO d6, solvent residues δH: 2.50ppm, δC 39.52 ppm) Mass spectra (MS, m/z) were recorded on a Xevo TQD (Z spray ESI, APCI) mass spectrometer by direct infusion and using positive electrospray ionization (ESI^+) or negative electrospray ionization (ESI^-).

^{18}F, as an aqueous solution, was purchased from the MD Anderson Cyclotron Radiochemical Facility (CRF) (Houston, TX). ^{77}Br as HBr or NH_4Br was purchased from University of Wisconsin or Washington University in St. Louis Cyclotron Facilities. ^{211}At was received from the Texas A&M University Cyclotron Institute (TAMU CI). Radioactivity was measured using a dose calibrator with specific calibration number per each nuclide (Capintec, Inc., model # CRC-15R, Florham Park, NJ).

Cell Lines.

MDA-MB-231, MDA-MB-436 were provided by the Cytogenetics and Cell Authentication Core at UT M.D. Anderson Cancer Center (Houston, TX); SUM149 and SUM1315 were purchased from BioIVT (Hicksville, NY); all the other cell lines were obtained from ATCC.

HeLa, MDA-MB-231 and MDA-MB-436 were grown in DMEM High Glucose medium with 10% FBS and 2 mM L-glutamine. Jurkat and MAVER-1 were grown in RPMI medium supplemented with 10% FBS and 2 mM L-glutamine. HL-60 were grown in IDME media plus 10% FBS and 2 mM L-glutamine. SUM149 were grown in F12 supplemented with 10% fetal bovine serum, 10 mM HEPES (Sigma, H3375), hydrocortisone 1 µg/ml (Sigma, H4001) and insulin 10 µg/ml (Sigma, I9279). SUM1315 were grown in F12 supplemented with 10% fetal bovine serum, 10 mM HEPES (Sigma, H3375), hydrocortisone 1µg/ml (Sigma, H4001), insulin 10 µg/ml (Sigma, I9279) and EGF 10 ng/ml (Sigma, E9644). All cells were maintained in a humidified atmosphere at 37℃ with 5% CO_2; medias were purchased from Gibco and supplied with the specific additives based on cell specification materials.

Chemical Synthesis.

1-methyl-1H-1,2,4-triazole-5-carbaldehyde (3): 1-methyl-1H-1,2,4-triazole (60.2 mmol) was dissolved in dry diethyl ether (Et_2O) and dry tetrahydrofuran (THF) in a three neck round bottom flask equipped with rubber septa under N_2 atmosphere. The solution was cooled to -45℃ then nBuLi was added dropwise over 25 minutes. The reaction was stirred for 90 minutes then cooled down to -78℃, dimethyl formamide (DMF) was added and the reaction was stirred overnight at room temperature. The reaction was quenched with HCl 1.5 M, the layers separated, and the organic phase extracted three times with HCl 1.5M. The acidic pH of the combined aqueous phases was adjusted (pH 9) with Na_2CO_3 and the aqueous phase was extracted three times with DCM. The organic phase was washed with brine and dried over Na_2SO_4. The crude mixture was purified using flash chromatography to provide **(3)** as a yellowish oil (yield 35.6%).

3: ^{1}H NMR (600 MHz, CDCl3) δ 10.0 (s, 1H), 8.0 (s, 1H), 4.2 (s, 3H).

Methyl-2-(4-bromophenyl)-7-fluoro-3-(1-methyl-1H-1,2,4-triazol-5-yl)-4-oxo-1,2,3,4-tetrahydro-quinoline-5-carboxylate (7) was synthetized using p-bromobenzaldehyde in a methodology previously published[1] and obtained as trans racemic mixtures [(8S,9R)/(8R,9S)] and a pale yellow powder (**Scheme S1A**).

7: ^{1}H NMR (500 MHz, DMSO-*d6*) δ 12.36 (s, 1H), 7.81 (s, 1H), 7.54-7.52 (m, 2H), 7.43-7.41 (m, 2H), 7.09-7.07 (dd, *J*= 2.5, 9.0 Hz, 1H), 6.93-6.91 (dd, *J*= 2.5, 8.9 Hz, 1H), 5.01 (d, *J*= 3.5 Hz, 2H), 3.69 (s, 3H).

^{13}C NMR (125 MHz, DMSO-*d6*) δ 166.50 (s), 164.39 (s), 159.37 (s), 152.32 (s), 150.79 (s), 148.85 (s), 141.39 (s), 138.99 (s), 131.78 (s), 130.76 (s), 121.95 (s), 111.78 (s), 103.29 (d), 99.02 (d), 59.10 (s), 42.77 (s), 35.39 (s).

MS (ESI) *m/z*: 439.70 $[M-H]^-$

Synthesis of 8-(4-bromophenyl)-5-fluoro-9-(1-methyl-1H-1,2,4-triazol-5-yl)-2,7-bis((2-(trimethylsilyl) ethoxy) methyl)-2,7,8,9-tetrahydro-3H-pyrido[4,3,2-de] phthalazin-3-

one (8) (Scheme S1A): To a round bottom flask under Ar atmosphere containing methyl 2-(4-bromophenyl)-7-fluoro-3-(1-methyl-1H-1,2,4-triazol-5-yl)-4-oxo-1,2,3,4-tetrahydroquinoline-5-carboxylate (**7**, 412.9 mg, 0.94 mmol) at 0°C, in anhydrous THF (12 ml), sodium hydrate (180.5 mg, 7.52 mmol) was added portion wise under Ar atmosphere. The reaction was stirred for 45 min at 0°C before warming up to room temperature upon which 2-(trimethylsilyl) ethoxymethyl chloride (SEM-Cl) was added dropwise over 25 min. The mixture was stirred overnight at room temperature. The reaction was quenched with water and extracted with DCM (4 x 35 ml). The organic layer was washed with brine (4 x 40 ml), then dried with Na_2SO_4 and the solvent was removed under vacuum. The crude material was purified using flash chromatography (% MeOH in DCM, 0% for 3CV, 0-20% in 15CV, 50% for 4CV) on a Biotage Isolera One system. 8-(4-bromophenyl)-5-fluoro-9-(1-methyl-1H-1,2,4-triazol-5-yl)-2,7-bis((2-(trimethylsilyl)ethoxy)methyl)-2,7,8,9-tetrahydro-3H-pyrido[4,3,2-de]phthalazin-3-one (**8**) was obtained as a yellow solid (536.1 mg, 82%)

8: ^{1}H NMR (500 MHz, chloroform-*d*) δ 7.73 (s, 1H), 7.46-7.44 (dd, *J*= 2.3, 8.7Hz, 1H), 7.41-7.39 (m, 2H), 7.12-7.10 (dd, *J*= 2.5,11.0 Hz, 1H), 7.07-7.05 (m, 2H), 5.43-5.36 (q, 2H), 5.21 (d, *J*= 3.7 Hz, 1H), 4.88-4.78 (q, 2H), 4.53 (d, *J*= 4.0 Hz, 1H), 3.94 (s, 3H), 3.45-3.30 (m, 4H), 0.82 (t, *J*= 8.17 Hz, 4H), 0.00018 (s, 18H).

^{13}C NMR (125 MHz, chloroform-*d*) δ 168.80 (s), 166.81 (s), 160.76 (d), 154.15 (s), 151.96 (s), 139.43 (s), 139.10 (s), 133.66 (d), 131.72 (s), 129.94 (d), 123.83 (s), 113.43 (s), 105.84 (d), 104.09 (d), 84.32 (s), 80.43 (s), 68.72 (s), 67.45 (s), 66.13 (s), 61.66 (s), 44.66 (s), 37.59 (s), 19.43 (d), 1.44 (s).

MS (ESI) *m/z*: 701.71 $[M+H]^+$

Synthesis of the final precursor 5-fluoro-9-(1-methyl-1H-1,2,4-triazol-5-yl)-8-(4-(4,4,5,5-tetramethyl-1,3,2-dioxaborolan-2-yl)phenyl)-2,7-bis((2-(trimethylsilyl)ethoxy) methyl)-2,7,8,9-tetrahydro-3H-pyrido[4,3,2-de]phthalazin-3-one (1) (Scheme S1A): To an oven-dried round bottom flask under argon atmosphere, compound (**8**) (400 mg, 0.57 mmol), bis(pinacolato)diboron (327.6 mg, 1.3 mmol), Pd(dppf)Cl_2 (14.6 mg, 0.02 mmol) and potassium acetate (176.7 mg, 1.8 mmol) were added. The flask was backfilled with argon and placed in a heating block at 90°C, upon which degassed DMF (3 ml) was added and the reaction was stirred overnight. The reaction mixture was passed through a celite plug, then diluted with water and extracted with EtOAc (4 x 30ml). The organic layer was washed with saturated lithium chloride (3 x 40ml) than dried over Na_2SO_4. Excess solvent was removed *in vacuo*, then 5%DMF in methanol were added to precipitate the radiolabeling precursor (**1**) as a white solid (113 mg, 26.5%).

1: ^{1}H NMR (500 MHz, chloroform-*d*) δ 7.81 (s, 1H), 7.79-7.78 (d, *J*= 8.0 Hz, 2H), 7.54-7.52 (dd, *J*= 2.4 Hz, 8.3 Hz, 1H), 7.26-7.25 (d, *J*= 8.0 Hz, 2H), 7.22-7.20 (dd, *J*= 2.2 Hz, 11.0 Hz,

1H), 5.48-5.44 (q, 2H), 5.31 (d, *J*= 4.3 Hz, 1H), 4.95-4.80 (q, 2H), 4.46 (d, *J*= 4.6 Hz, 1H), 3.98 (s, 3H), 3.68-3.65 (m, 2H), 3.49-3.37 (m, 2H), 1.40 (s, 12H), 0.88 (t, *J*= 8.2 Hz, 4H), 0.00010 (s, 18H).

^{13}C NMR (125 MHz, chloroform-*d*) δ 168.76 (s), 166.75 (s), 160.86 (d), 154.36 (s), 151.97 (s),147.69 (d), 143.09 (s), 139.49 (s), 136.94 (s), 131.68 (s), 127.65 (s), 113.70 (s), 105.86 (d), 103.99 (d), 85.42 (s), 83.55 (s), 80.46 (s), 68.66 (s), 67.40 (s), 66.73 (s), 44.90 (s), 37.43 (s), 26.28 (s), 19.45 (d), 1.44 (s).

MS (ESI) *m/z*: 749.79 $[M+H]^+$

Synthesis methyl 7-fluoro-3-(1-methyl-1H-1,2,4-triazol-5-yl)-4-oxo-2-(4-(4,4,5,5-tetramethyl-1,3,2-dioxaborolan-2-yl)phenyl)-1,2,3,4-tetrahydroquinoline-5-carboxylate (2): Compound (**2**) was synthetized using 4-(4,4,5,5-tetramethyl-1,3,2-dioxaborolan-2-yl)benzaldehyde in a methodology previously published[1] and obtained as trans racemic mixtures [(8S,9R)/(8R,9S)] of a yellow powder (**Scheme S1B**).

2: ^{1}H NMR (500 MHz, DMSO-*d6*) δ 12.34 (s, 1H), 7.79 (s, 1H), 7.71 (s, 1H), 7.61-7.60 (m, 2H), 7.46-7.45 (m, 2H), 7.08-7.06 (dd, *J*= 2.9, 9.2 Hz, 1H), 6.94-6.92 (dd, *J*= 2.5, 11.2 Hz, 1H), 5.01 (d, *J*= 3.5 Hz, 2H), 3.64 (s, 3H), 1.28 (s, 12H)

^{13}C NMR (125 MHz, DMSO-*d6*) δ 166.38 (s), 164.41 (s), 159.38 (s), 152.44 (s), 150.70 (s),142.82 (s), 141.48 (s), 134.88 (s), 134.58 (s), 128.01 (s), 111.78 (s), 103.46 (d), 99.07 (d), 84.20 (s), 59.77 (s), 49.07 (s), 35.27 (s), 25.11 (s).

MS (ESI) *m/z*: 489.48 $[M+H]^+$

Synthesis of 5-fluoro-8-(4-iodophenyl)-9-(1-methyl-1H-1,2,4-triazol-5-yl)-2,7,8,9-tetrahydro-3H-pyrido[4,3,2-de]phthalazin-3-one (11) (**Scheme S1C**)**:** Compound (**11**) was synthetized using p-iodobenzaldehyde in a methodology previously published[1] and obtained as trans racemic mixtures [(8S,9R)/(8R,9S)] of a white powder.

11: ^{1}H NMR (500 MHz, DMSO-*d6*) δ 12.36 (s, 1H), 7.81 (s, 1H), 7.71-7.70 (m, 2H), 7.69 (s, 1H), 7.27-7.25 (m, 2H), 7.08-7.07 (dd, *J*= 2.5, 8.6 Hz, 1H), 6.92-6.90 (dd, *J*= 2.5, 11.08 Hz, 1H), 5.00 (d, *J*= 10.9 Hz, 2H), 3.69 (s, 3H).

^{13}C NMR (125 MHz, DMSO-*d6*) δ 166.50 (s), 164.39 (s), 159.37 (s), 152.32 (s), 150.79 (s), 148.85 (s), 141.39 (s), 138.99 (s), 131.78 (s), 130.76 (s), 121.95 (s), 111.78 (s), 103.29 (d), 99.02 (d), 59.10 (s), 42.77 (s), 35.39 (s).

MS (ESI) *m/z*: 489.08 $[M+H]^+$

A

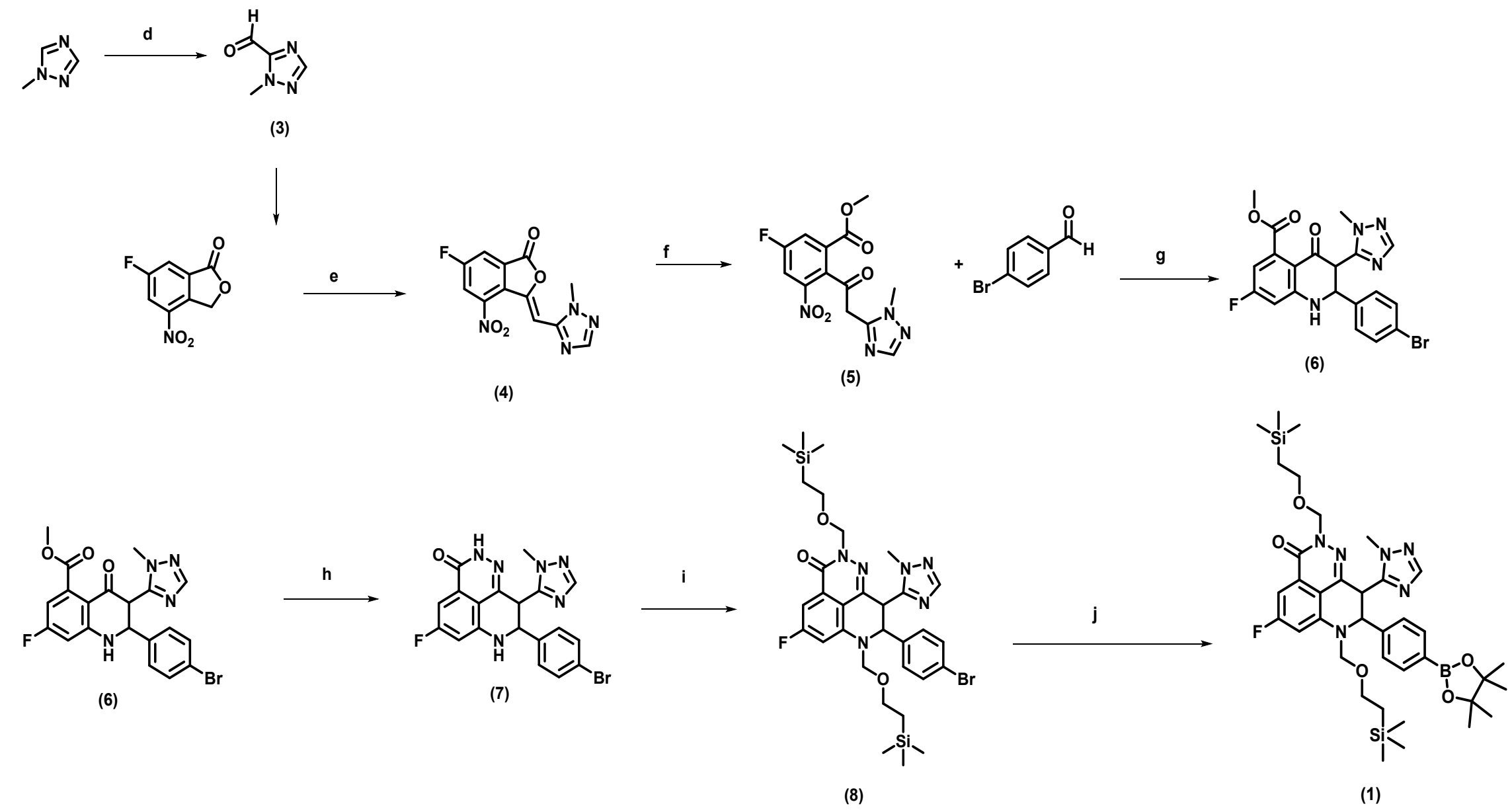

B

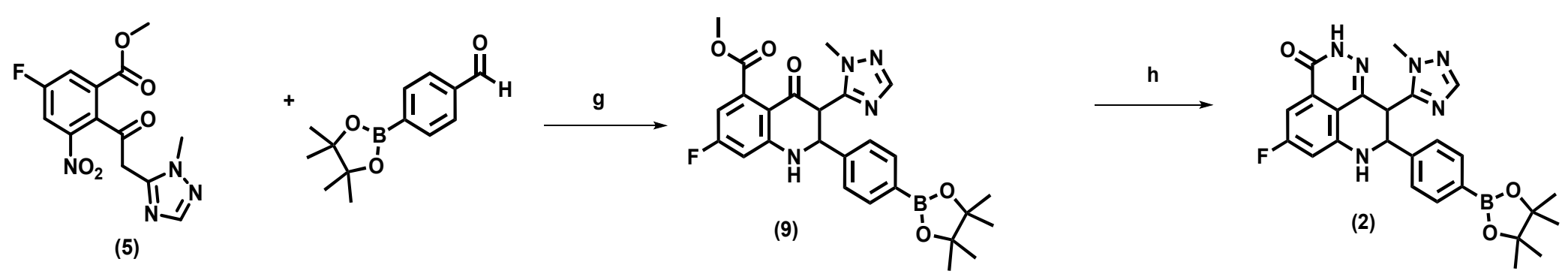

C

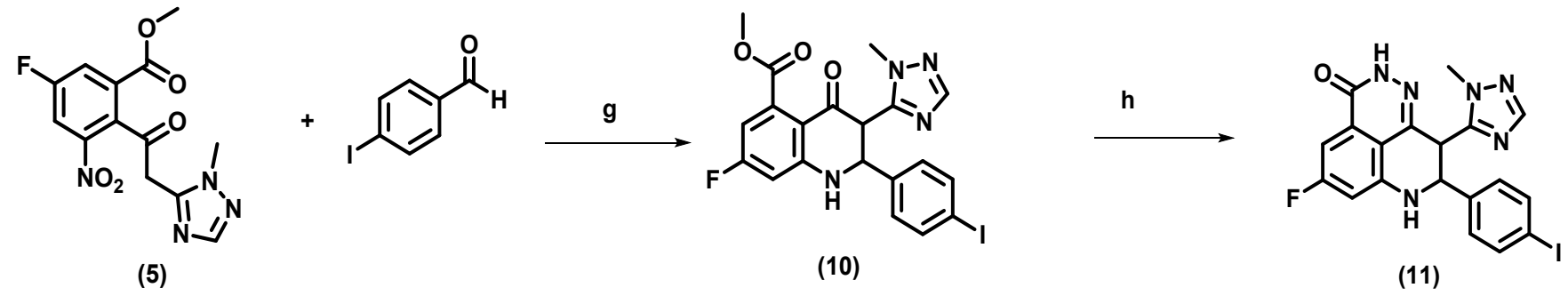

Scheme S1: (A) Full synthetic scheme for the fully protected precursor (**1**), (**B)** the unprotected precursor (**2**), and **(C)** the I-TZ standard (**11**). Reaction conditions: (d) 1) nBuLi, THF/Et_2O, -45°C, 90 min, 2) -78°C, DMF, rt ON. (e) Et_3N, Ac_2O, 2-methyl-THF, reflux, (f) AcOH, MeOH, 56°C, 6h. (g) 1) $TiCl_3$, THF, MeOH, 15 min at rt, 2) 60°C for 6h. (h) hydrazine, MeOH, 10h. (i) 1) NaH, 0°C, 45 min THF, 2) SEM-Cl, ON 0°C to rt. (j) [Pd(dppf)$_2$Cl$_2$], KAcO, bis(pinacolato)diboron, DMF, 90°C ON.

Table S1. Binding Interactions from Docking and Surface Rendering of TZ in the PARP1 Binding Pocket.

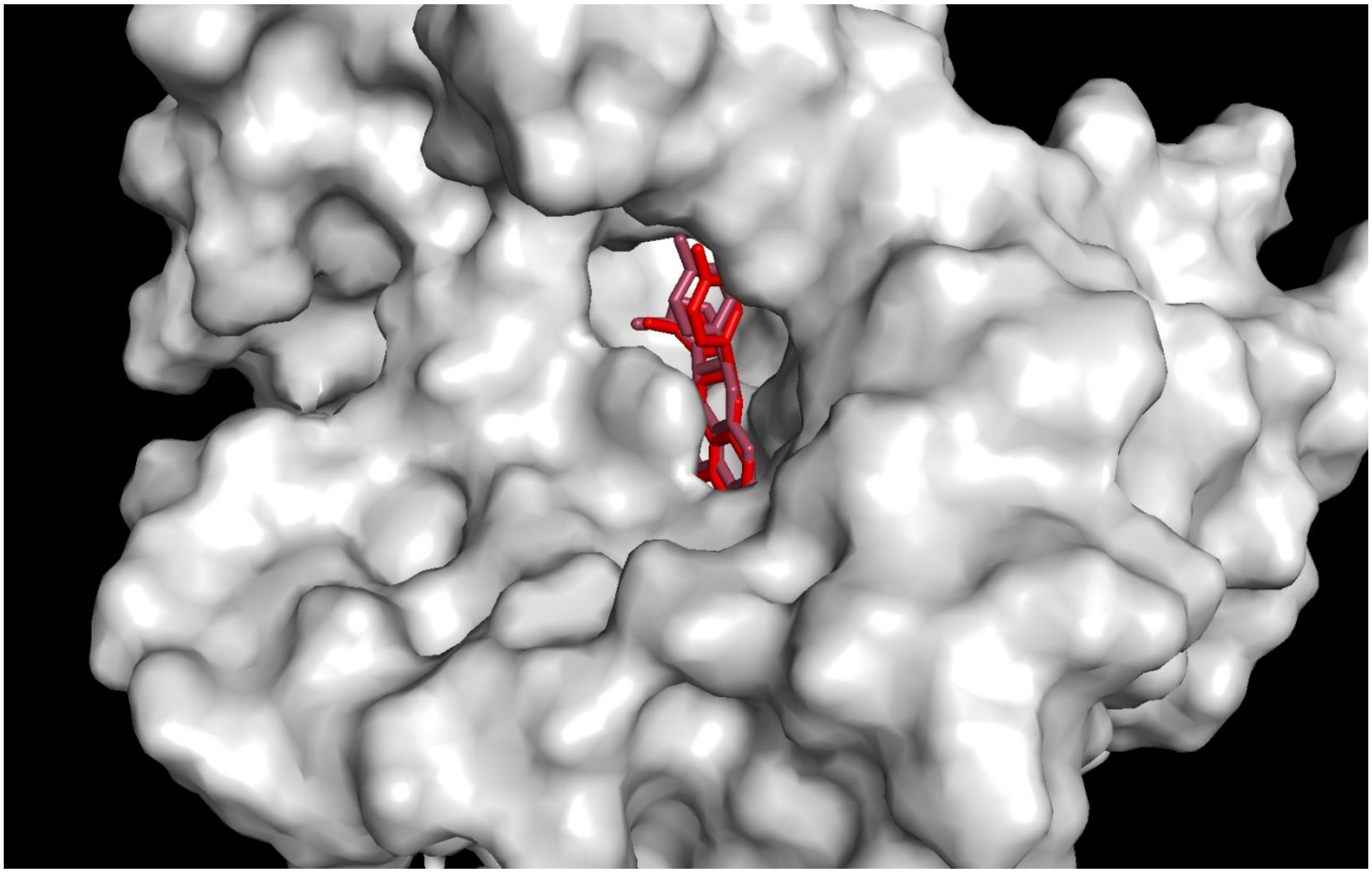

Ligand	Affinity (kcal/mol)	Hydrogen bond	Hydrophobic interaction	Π-Π stacking	Halogen bond	Others
Talazoparib	-10.83	TYR896<br>GLU988<br>GLY863<br>SER904	TYR896	TYR889<br>TYR907	GLN759	His862
Cl-Talazoparib	-8.686	GLU763<br>SER864	TYR896<br>TYR889<br>TYR907	TYR889<br>TYR907	SER904	
Br-Talazoparib	-9.108	GLU763	TYR896	TYR889	SER904	

		SER864	TYR889	TYR907	
			TYR907		
I-Talazoparib	-8.98	GLU763	TYR896	TYR889	SER904
		SER864	TYR889	TYR907	
			TYR907		
At-Talazoparib	-8.684	GLU763	TYR896	TYR889	
		SER864	TYR889	TYR907	
			TYR907		

Radiolabeling of Halo-Talazoparib Series.

Synthesis of 5-[^{18}F] fluoro-8-(4-fluorophenyl)-5-fluoro-9-(1-methyl.1H.1,2,4-triazol-5-yl)-2,7,8,9-tetrahydro-3H-pyrido[4,3,2-de]phthalazin-3-one (^{18}F-Talazoparib, [^{18}F]-12):

Radiosynthesis was performed on a TracerLab FX (General Electric Healthcare, Münster, Germany) automatic module. [^{18}F]fluoride was obtained as an aqueous solution from the MD Anderson Cyclotron Radiochemical Facility (CRF). [^{18}F]Fluoride was adsorbed on an ion exchange cartridge (pre-conditioned Sep-PAK® Light QMA Cartridge, ABX GmbH, Radeberg, Germany). [^{18}F]fluoride was flushed into the reaction vial with a potassium carbonate and Kryptofix 2.2.2. water/CH_3CN solution (700 µL; 52.8 mg of K_2CO_3, 240.1 mg of K_{222}, 4 mL of water, 16 mL of CH_3CN). The solution was dried under vacuum and under nitrogen flow at 60°C for 2 min. 500 µL of dry CH_3CN was added and then the mixture was azeotropically dried at 120°C for an additional 3 min. Synthesis of ^{18}F-Talazoparib was first carried out by adding the N-silanyl–protected boronic pinacol ester precursor (**1**, 5-10 mg) and [$Cu(OTf)_2(Py)_4$] (15-17 mg) in dry DMF (600 µL) to the dried [^{18}F]fluoride. Air was allowed to enter into the reactor by leaving vial 3 open during the setup of the instrument and closed before starting the synthesis. The mixture was stirred at 120°C for 20 min, cooled down at 80°C to generate the protected intermediate, then HCl 4M (1 mL) was added for the deprotection, yielding crude product [^{18}F]**-12**. The mixture was stirred at 120°C for 10 min, cooled down at 30°C and diluted with water (3 mL). The crude was purified by semi preparative HPLC (Luna 5 µm C18(2) 100 Å, 250 x 10

mm) eluting with 30% MeCN/water (0.085% H_3PO_4) [rt=17 min]. For final dispensation, the desired radioactive product was collected into a TracerLab collection flask pre-filled with water (26 mL). The solution was loaded onto a Sep-Pak C18 Plus Light Cartridge, 130 mg Sorbent (Sep-PAK®, Waters, Milford, USA). Cartridges were washed with 8 mL of water, dried under nitrogen and eluted with ethanol (1 mL). The overall synthesis time was approximately 100 min. Activity was determined by dose calibrator and a sample taken for quality control (QC). QC was performed by analytical radio-HPLC (Agilent 1260 infinity II equipped with an in-line LabLogic flow HPLC radio detector) on a C18 column (Waters, XBridge C18 column, 4.6 x 250 mm, 3.5 µm), using a water (0.1% (v/v) TFA) and CH_3CN (0.1% (v/v) TFA) gradient (20% B → 40% in 5 min, 40% B for 10 min, 40% B → 95% B in 6 sec, 95% B for 6 min) at a flow rate of 1 mL/min. Under these conditions, ^{18}F-Talazoparib presented a retention time of ~ 9 minutes . The identity of the radiolabeled compound was confirmed by co-elution of the original cold standard (Selleckchem, Houston, TX, USA).

Synthesis of 5-[^{77}Br]8-(4-bromophenyl)-5-fluoro-9-(1-methyl-1H-1,2,4-triazol-5-yl)-2,7,8,9-tetrahydro-3H-pyrido[4,3,2-*de*]phthalazin-3-one, (^{77}Br-Talazoparib analog, [^{77}Br]-13):

Synthesis of ^{77}Br-Talazoparib was performed manually in a warm-cell and carried out by dissolving the N-silanyl–protected boronic pinacol ester precursor (**1**, 1.3-4.5 mg) and [$Cu(OTf)_2(Py)_4$] (0.9-1.3mg) in dry DMSO (500 µL) to a dry sealed vial. A variable amount of [77Br]HBr/NH_4Br (300-600 µCi) was assed to the reactor and the mixture was heated and stirred at 120°C for 20 min to generate the protected intermediate. The mixture was cooled down to ~50°C, then 4M HCl (0.5-1 ml) was added for the deprotection of the intermediate. The reaction mixture was stirred at 120°C for 10 min and successively cooled down to room temperature. The crude was purified via semi-preparative HPLC (Econosil C18 10µm 250x10 mm) eluted with 40% ACN/Water + 0.1% TFA [rt = 12min]. The purified peak was diluted with water (35 ml) and trapped onto a Sep-Pak C18 Plus Light Cartridge, 130 mg Sorbent (Sep-PAK®, Waters, Milford, USA). Cartridge was washed with 8 mL of water, dried under air and eluted with ethanol (1 mL). The overall synthesis time was approximately 70 min. Activity was determined by dose calibrator and a sample taken for QC. QC was performed by analytical radio-HPLC (Agilent 1260 infinity II equipped with an in-line LabLogic flow HPLC radio detector) on a C18 column (Waters, XBridge C18 column, 4.6 x 250 mm, 3.5 µm), using a water (0.1% (v/v) TFA) and CH_3CN (0.1% (v/v) TFA) gradient (20% B → 40% in 5 min, 40% B for 10 min, 40% B → 95% B in 6 sec, 95% B for 6 min) at a flow rate of 1 mL/min. Under these conditions, ^{77}Br-Talazoparib presented a retention time of ~ 11 minutes. The identity of the radiolabeled compound was confirmed by co-elution of the Br-Talazopiarib analog cold standard previously synthetized.

Synthesis of 5-[^{211}At] 8-(4-astatophenyl)-5-fluoro-9-(1-methyl-1H-1,2,4-triazol-5-yl)-2,7,8,9-tetrahydro-3H-pyrido[4,3,2-*de*]phthalazin-3-one (^{211}At-Talazoparib analog, [^{211}At]-14):

Synthesis of ^{211}At-Talazoparib was performed manually in a warm-cell and carried out by dissolving the un-protected boronic pinacol ester precursor (**2**, 2.4 mg) in 200 µl of a 1:1 solution of acetonitrile and ethanol. Successively 0.5 mg of [Cu(OTf)2(Py)4] and 0.2 mg of 3,4,7,8-Tetramethyl-1,10-phenanthroline were dissolved in 100 µl of a 1:1 solution of methanol-ethanol and added to the precursor solution. A variable amount of ^{211}At in ethanol (0.25-1.1 mCi) was added to the reactor and to the final volume $NaOH_{MeOH}$ (after fresh standardization) was added to a final concentration of 0.1M. The mixture was stirred at room temperature for 10-15 min. The crude was diluted with 1 ml of water and purified via semi-preparative HPLC (Econosil C18 10µm 250x10 mm) eluted with 40% ACN/Water [rt = 17 min]. The purified peak was diluted with water (50 ml) and trapped onto an OASIS HLB Plus Light Cartridge, 100 mg Sorbent (Sep-PAK®, Waters, Milford, USA). Cartridge was washed with 8 mL of water, dried under air and eluted with ethanol (1 mL). The overall synthesis time was approximately 45 min. Activity was determined by dose calibrator and a sample taken for QC. QC was performed by analytical radio-HPLC (Agilent 1260 infinity II equipped with an in-line LabLogic flow HPLC radio detector) on a C18 column (Waters, XBridge C18 column, 4.6 x 250 mm, 3.5 µm), using a water (0.1% (v/v) TFA) and CH_3CN (0.1% (v/v) TFA) gradient (20% B → 40% in 5 min, 40% B for 10 min, 40% B → 95% B in 6 sec, 95% B for 6 min) at a flow rate of 1 mL/min. Under these conditions, ^{211}At-Talazoparib presented a retention time of ~ 14 minutes. The identity of the radiolabeled compound was confirmed by co-elution of the I-Talazoparib analog cold standard previously synthetized.

Shelf and 4°C Stability of Radio-Halo-Talazoparib and Precursors.

The various radio-TZ derivatives were diluted in PBS/10% ethanol and stored at room temperature or 4°C. An aliquot of each sample was taken following a specific timetable and the presence of side species was studied by analytical radio-HPLC (Agilent 1260 infinity II equipped with an in-line LabLogic flow HPLC radio detector) on a C18 column (Waters, XBridge C18 column, 4.6 x 250 mm, 3.5 µm), using a water (0.1% (v/v) TFA) and CH_3CN (0.1% (v/v) TFA) gradient (20% B → 40% in 5 min, 40% B for 10 min, 40% B → 95% B in 6 sec, 95% B for 6 min) at a flow rate of 1 mL/min.

Although systematic shelf stability of precursors was not fully evaluated at this stage of development, precursors were stored in a desiccator at 4°C in the dark, and this was sufficient to enable labeling for several months. After 1 year, precursors required re-purification by LC-MS. Precursor **1** is similar to precursors of an agent in active phase 2 clinical trials produced under cGMP (e.g., ABX, Germany) and supporting an automated cGMP radiosynthesis process (NCT07325253).

Calculations for Molar Activity for Compounds.

Below is the description of the HPLC method, run time, and example AUC calculation (TZ) for determination of MA.

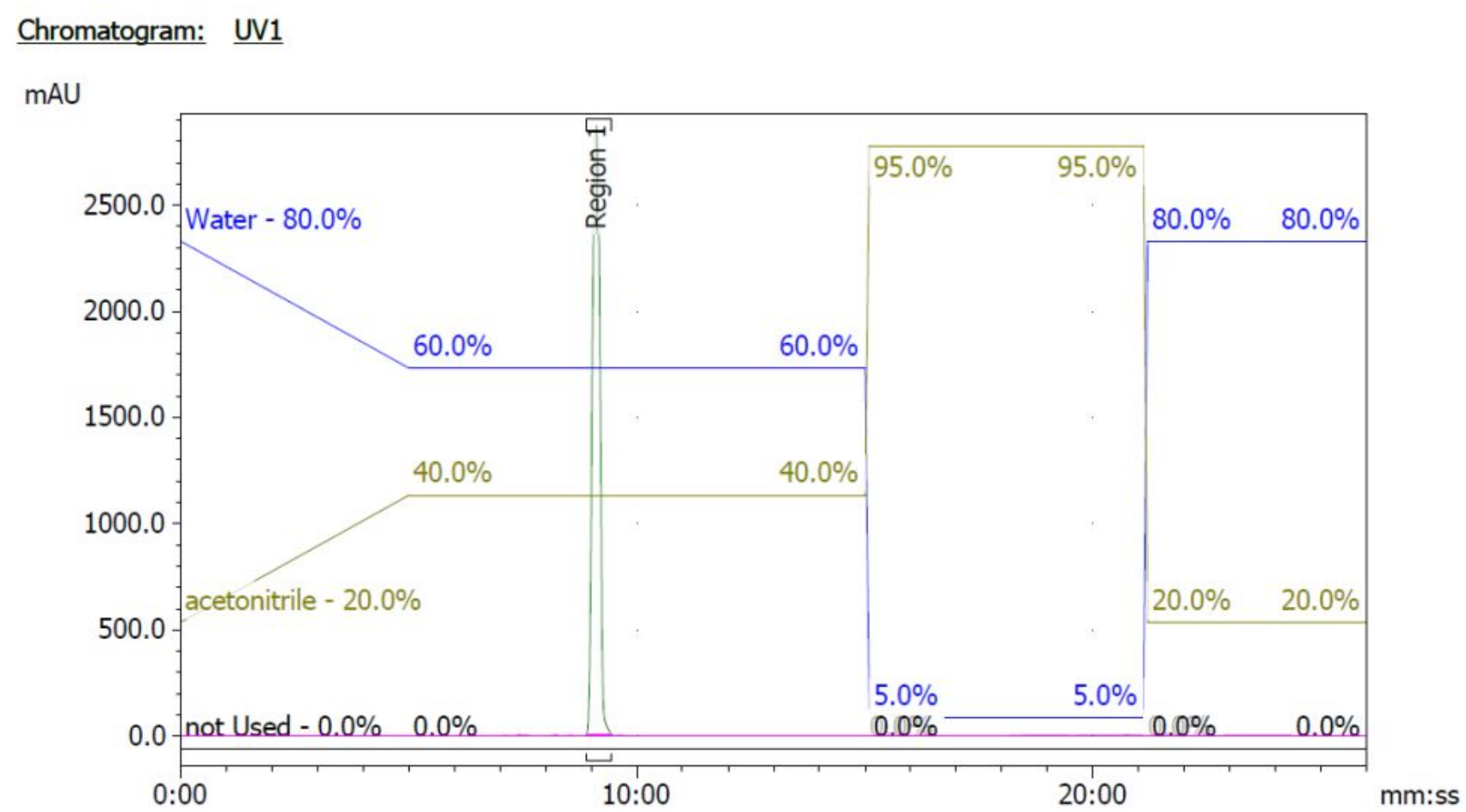

Regions: UV1 Detector:

Name	Start (mm:ss)	End (mm:ss)	Retention (mm:ss)	Area (mAU·s)	%ROI (%)	%Total (%)
Region 1	8:54	9:27	9:07	30384.5	100.00	92.25
1 Peak				30384.5	100.00	92.25

Calibration Curve for Enantio-Pure TZ.

This calibration curve was leveraged for MA calculations for ^{77}Br-TZ and ^{211}At-TZ assuming only minor changes to system responsiveness of UV absorption for halogen substitutions. Example spreadsheet calculations are included below for how stock concentration, specific activity and molar activity were calculated. Calculations were then based upon simple dilution from addition of stock solutions and use of appropriate molecular weights.

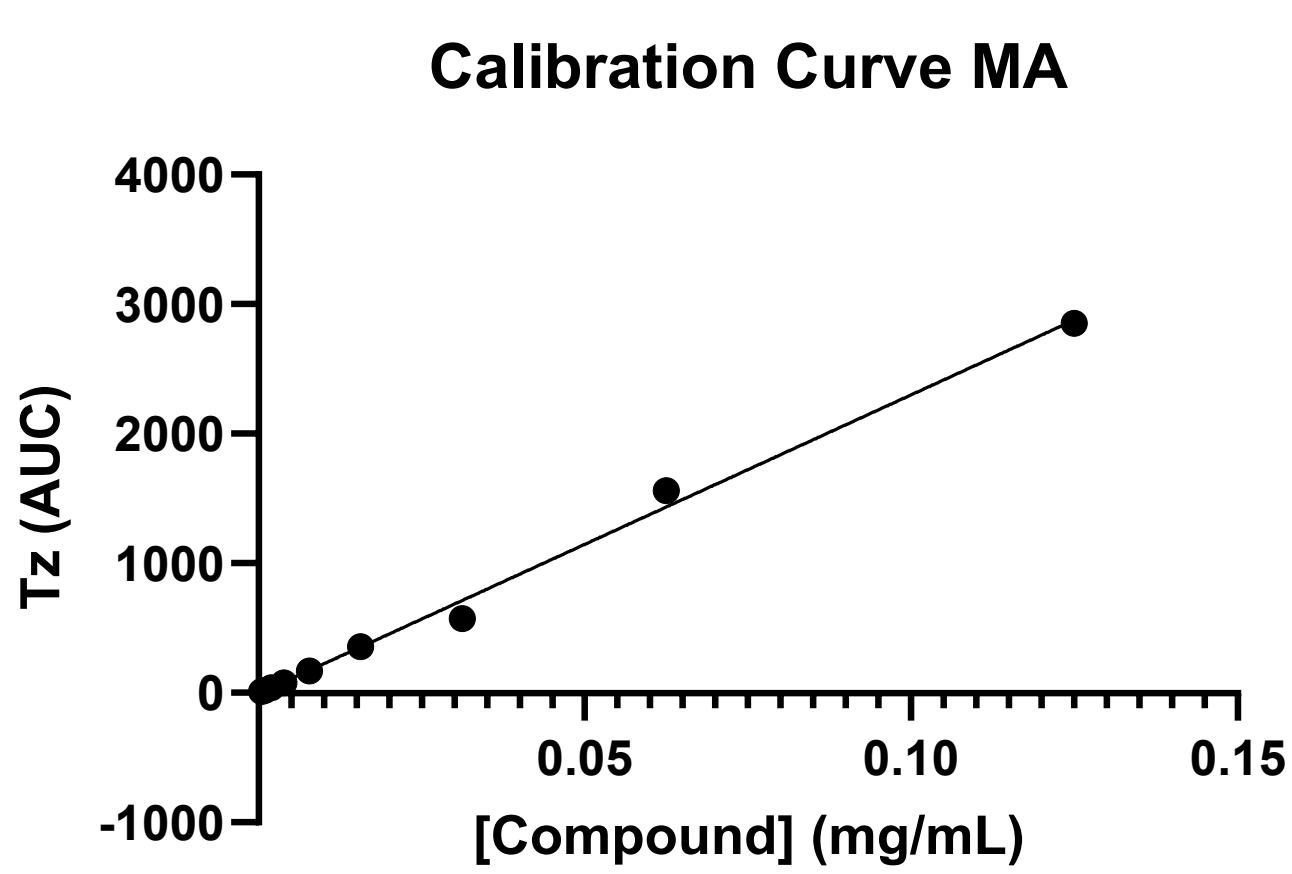

Straight line	
Best-fit values	
YIntercept	-7.155
Slope	23078
95% CI (profile likelihood)	
YIntercept	-46.92 to 32.61
Slope	22084 to 24071
Goodness of Fit	
Degrees of Freedom	11
R squared	0.9958
Sum of Squares	35904
Sy.x	57.13

^{18}F-TZ

18F-TZ EXPT NUMBER	Stock volume (ml)	Activity (µCi)	Act volume (ml)	activity prelevated (µCi)	injected activity (µCi)	Conversion Factor	Activit injected (GBq)	Area (mAu)	Conc (mg/ml)	injected volume (ml)	injected mass (mg)	**As (GBq/mg)**	MM (g/mol)	injected mol (mmol)	injected µmol	**Am (GBq/µmol)**
RM1029	0.1	189	0.05	94.5	47.25	3.70E-05	0.00174825	2.4	0.000414031	0.1	4.14031E-05	42.22513187	380.35	1.08855E-07	0.000108855	16.06032891
RM1030	0.2	212	0.05	53	26.5	3.70E-05	0.0009805	2.2	0.000405364	0.1	4.05364E-05	24.18811224	380.35	1.06577E-07	0.000106577	9.19994849
RM1031	0.2	268	0.05	67	33.5	3.70E-05	0.0012395	5	0.000526692	0.1	5.26692E-05	23.53367421	380.35	1.38476E-07	0.000138476	8.951032985

^{77}Br-TA

77Br-TZ EXPT NUMBER	Stock volume (ml)	Activity (µCi)	Act volume (ml)	activity prelevated (µCi)	injected activity (µCi)	Conversion Factor	Activit injected (GBq)	Area (mAu)	Conc (mg/ml)	injected volume (ml)	injected mass (mg)	**As (GBq/mg)**	MM (g/mol)	injected mol (mmol)	injected µmol	**Am (GBq/µmol)**
5701	1	78	0.05	3.9	1.95	3.70E-05	7.22E-05	296.7	0.013166436	0.1	0.001316644	0.05479843	441.26	2.98383E-06	0.002983827	0.024180355
5702	1	113	0.05	5.65	2.825	3.70E-05	0.000104525	516.4	0.022686325	0.1	0.002268632	0.046074012	441.26	5.14126E-06	0.00514126	0.020330619
5703	1	130	0.05	6.5	3.25	3.70E-05	0.00012025	41.6	0.002112618	0.1	0.000211262	0.569198954	441.26	4.78769E-07	0.000478769	0.25116473

^{211}At-TA

211At-TZ EXPT NUMBER	Stock volume (ml)	Activity (µCi)	Act volume (ml)	activity prelevated (µCi)	injected activity (µCi)	Conversion Factor	Activit injected (GBq)	Area (mAu)	Conc (mg/ml)	injected volume (ml)	injected mass (mg)	**As (GBq/mg)**	MM (g/mol)	injected mol (mmol)	injected µmol	**Am (GBq/µmo**
5011 1-2	0.6	90.6	0.05	7.55	3.775	3.70E-05	0.000139675	26.5	0.001458315	0.1	0.000145832	0.957783286	571.36	2.55236E-07	0.000255236	0.547239058
5011 3-4	0.6	121.7	0.05	10.14166667	5.070833333	3.70E-05	0.000187621	5.4	0.000544025	0.1	5.44025E-05	3.448756345	571.36	9.52157E-08	9.52157E-05	1.970481426
5013 1-2	1	590	0.05	29.5	14.75	3.70E-05	0.00054575	29.5	0.001588309	0.1	0.000158831	3.436043787	571.36	2.77987E-07	0.000277987	1.963217978

Cell Uptake of Radioactive Halo-Talazoparib in Different Cell Lines.

Cellular uptake of ^{18}F-TZ at 37℃, uptake at 4℃, and cold blocking were studied in Hela, SUM149, SUM1315, MDA-MB-436, MDA-MB-231 cells. All experiments were performed in quadruplicate in a 24-well plate format. For cell uptake studies, $1x10^5$ cells were equilibrated in MEBSS (1 ml), and then ^{18}F-Talazoparib was added (~1 µCi ~37 kBq or 2.5 µL maximum volume) to each well and incubated at 37°C for 120 min. Experiments that abrogated metabolism were performed at 4°C, a temperature at which cellular membranes become less fluid and permeability low. For cold blocking experiments, cells were preincubated for 30 min at 37°C with excess non-radioactive Talazoparib (100 nM, Selleckchem S7048). After 120 min, an aliquot of the cell media (200 µl) was obtained for gamma counting, the remaining media removed, and cells washed with ice-cold MEBSS. Cells were then lysed with 1% SDS, 10 mM sodium borate for 30 min at room temperature. Radioactivity contained within the aliquots of cell media and cell lysates were measured using a WIZARD2 2480 automatic gamma counter (ParkinElmer). Protein contents were quantified using BCA protein assay kits (ThermoFisher Scientific) according to the manufacture's protocol using bovine serum albumin (BSA) as the protein standard. Cell net uptake values of ^{18}F-TZ were normalized to protein content and expressed as a cellular/extracellular tracer ratio $((cpm_{cells}/\text{mg protein})/(cpm_{media}/\text{mL}))$. This process was repeated for the relevant radiolabeled compounds.

Calculation of "In"-to-Out Ratios for ^{77}Br for Potential Dosimetry Estimations.

Published measurements of mg protein per cell [2] and volume per cell for HeLa cells [3] enable conversion of cell-associated counts to cellular dosimetry estimates. Total cell-associated counts were considered 'In' the cell. For the other cell lines, In-to-Out estimates from further extrapolations are contained below.

Constants

HeLa cells pg/cell	source	mg/pg	HeLa cells mg/cell
166.3333333	PMID: 5434280	1.00E-09	1.66E-07
MDA-MB-436 (ug/10^6 cells)	source	(mg/cell)/(ug/10^6 cells)	MDA-MB-436 cells mg/cell
220	https://research.fredhutch.org/paulovich/en/typical-protein-yields.html	0.000000001	2.20E-07
No published estimates for rest so estimated based upon HeLa and MDA-MB-436 and median posted of all cell lines (ug/10^6)	source	(mg/cell)/(ug/10^6 cells)	Others
200	https://research.fredhutch.org/paulovich/en/typical-protein-yields.html	0.000000001	2.00E-07

From 77Br Uptake Experiment	Hela	SUM149	SUM1315	MDA-MB-436	MDA-MB-231
Number of values	4	4	4	4	4
Minimum	0.06615	0.05989	0.02193	0.05008	0.02965
Maximum	0.09556	0.1291	0.1924	0.09958	0.08223
Range	0.0294	0.06921	0.1705	0.04949	0.05258
Mean (cpm/mg)/(cpm/mL) @ 40 nM	0.0844	0.08842	0.08928	0.07416	0.05675
Std. Deviation	0.01352	0.03156	0.07347	0.02079	0.02433
Std. Error of Mean	0.006761	0.01578	0.03673	0.01039	0.01217
Coefficient of variation	16.02%	35.69%	82.29%	28.03%	42.87%
**Mean**					
(cpm/cell)/(cpm/mL) or (mL/cell)	1.40E-08	1.77E-08	1.79E-08	1.63E-08	1.14E-08
~Volume of a tumor cell (Hela as model for solid tumor) (mL/cell)	2.43E-06	2.43E-06	2.43E-06	2.43E-06	2.43E-06
**Derived average whole cell In/Out Ratio @40 nM**	0.005789086	7.29E-03	7.36E-03	6.73E-03	4.68E-03
77Br cell killing Assay (assume saturating in to out ratio holds) and integrate an exponential exposure for 72 hr for 0.072 GBq/L concentration in media, and 2.053*10^5 s as half life for 77Br (Bq*s/mL) multiplied by above "in" to out ratio (Bq*s/mL)	71997430.63	90693417.39	91575529.35	83673447.38	58209131.84
**Multiply by volume of cell above (Bq*s/cell)**	1.75E+02	2.20E+02	2.22E+02	2.03E+02	1.41E+02

Western Blot Analysis.

Cell lysates were prepared as indicated following incubation in lysis buffer plus protease/phosphatase inhibitors (1 mM PMSF, 10 µg/ml leupeptin, 10 µg/ml aprotinin and 1 mM Na_3VO_4). Cells were lysed for 30 min on ice and then centrifuged at 17,000 × g for 30 min at 4°C. Protein concentration was assessed using a bicinchoninic acid (BCA) protein assay (Thermo Fisher Scientific, #23,225). Equal amounts of protein were separated by 8–16% SDS-PAGE, transferred to PVDF membranes, and subjected to Western blotting using anti-mouse (Bio-Rad, #170–6515) and anti-rabbit (Bio-Rad, #172–1019) secondary antibodies and an ECL chemiluminescence reagent (Bio-Rad, #170–5061) using an Azure-c600 imaging system (Azure Biosystems). Anti-GAPDH antibody (AF5718) was purchased from Fisher Scientific; anti-PARP1 rabbit monoclonal antibody (46D11) was purchased from Cell Signaling and used for Western blot analysis.

Cell-Free PARP1 enzyme activity.

Preliminary assessments of Br-TZ and I-TZ inhibition of PARP1 activity were determined using a commercial PARP1 enzyme activity kit (R&D Systems; cat #4677-096-K).

qPCR Analysis of mRNA Expression.

Cells were incubated at 37°C, treated as described, and RNA was extracted with AurumTM total RNS mini kit (Biorad, #7326820) following the manufacturers protocol. cDNA was synthesized from 300 ng of RNA using the iTaqTM universal SYBR green one-step kit (Biorad, #172551). GAPDH primers were utilized as previously published (Proc Natl Acad Sci U S A. 2020 Aug 24;117(36):22378–22389. doi: 10.1073/pnas.2003537117).

***PARP1* Primers**

PrimerBank ID 156523967c1

Amplicon Size 136

	**Sequence** (5' –> 3')	**Length**	**Tm**	**Location**
Forward Primer	CGGAGTCTTCGGATAAGCTCT	21	60.8	5-25
Reverse Primer	TTTCCATCAAACATGGGCGAC	21	61.2	140-120

***PARP2* Primers**

PrimerBank ID 110825962c1

Amplicon Size 81

	**Sequence** (5' –> 3')	**Length**	**Tm**	**Location**
Forward Primer	GCCTTGCTGTTAAAGGGCAAA	21	61.7	214-234
Reverse Primer	TCCTTCACAATACACATGAGCC	22	60.0	294-273

***PARP3* Primers**

PrimerBank ID 158634481c2

Amplicon Size 88

	**Sequence** (5' –> 3')	**Length**	**Tm**	**Location**
Forward Primer	CCGCAGAGAAGCGCATAATC	20	61.3	119-138
Reverse Primer	GTGCAGTTGTAGTCCTCATACAC	23	60.6	206-184

Cell Viability Assay.

Cells were cultured as above and plated on day 0. ^{77}Br-TZ at a concentration of 5 μCi/mL or equal volume of ethanol (vehicle) were added to cells on day 0 and plates returned to a dedicated shielded cell culture incubator. At 72 hours post incubation, cell viability was determined with an SRB protein assay kit (Abcam, ab235935-100Kt).

Supporting Figures.

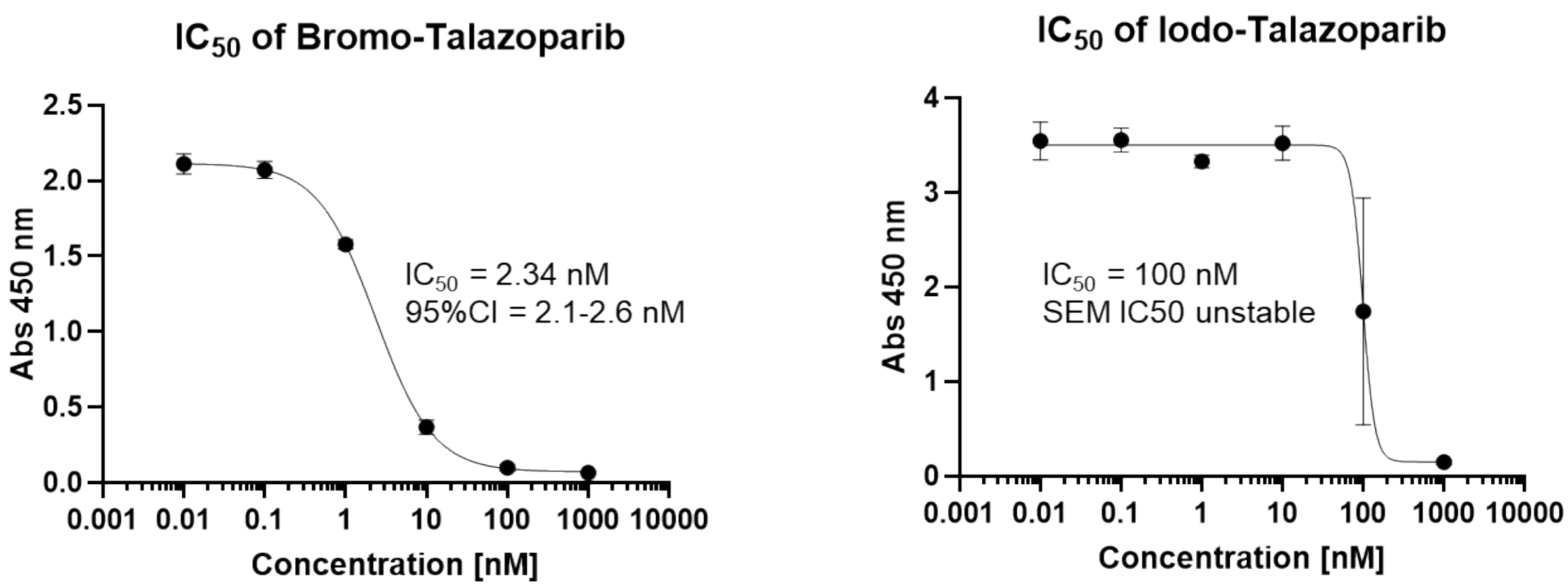

Figure S1. Preliminary Cell-Free PARP1 Inhibition Assay. Preliminary assessments of racemic Br-TZ and I-TZ inhibition of PARP1 activity were determined using a commercial PARP1 enzyme activity kit (R&D Systems; cat #4677-096-K). PARP1 enzyme inhibition IC_{50} values were determined with a variable slope four parameter fit (GraphPad Prizm). IC_{50} values for Br-TZ and I -TZ were 2.3 nM and 100 nM, respectively (R^2 >= 0.9). Quantitatively, due to the Hillslope, the 95% CI for Iodo-Tz cannot be calculated, however inspection of the graph indicates a range > 10 nM and < 1000 nM.

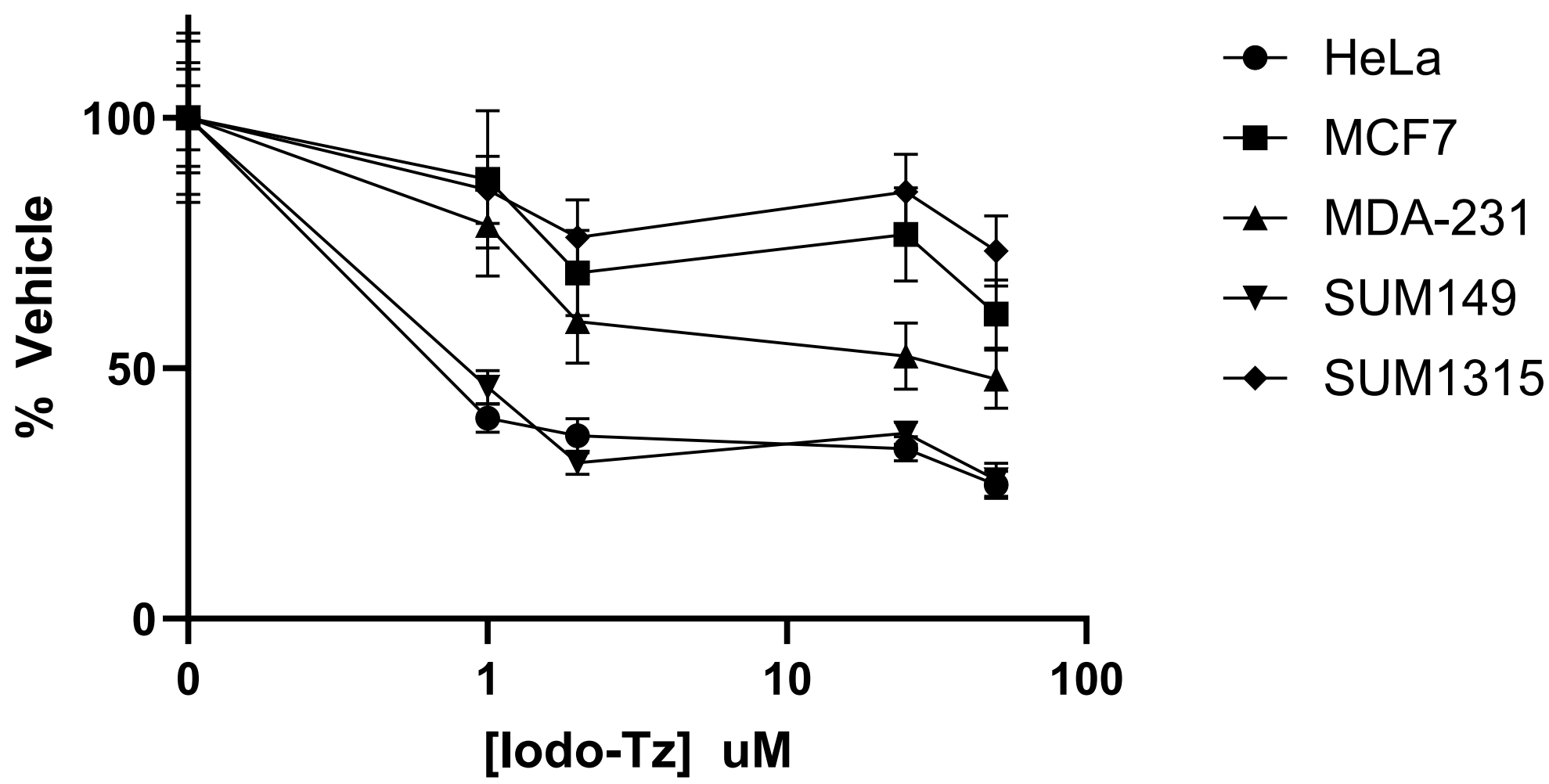

Figure S2. Preliminary Cell Cytotoxicity Assay for I-TZ. Cells were cultured as above and plated on day 0. Racemic I-TZ was added on day 0 to the media at the concentrations indicated and cell viability measured by SRB protein assay after 72 hours. Data are plotted as percent untreated control.

References.

(1) Wang, B.; Chu, D.; Feng, Y.; Shen, Y.; Aoyagi-Scharber, M.; Post, L. E. Discovery and Characterization of (8S,9R)-5-Fluoro-8-(4-fluorophenyl)-9-(1-methyl-1H-1,2,4-triazol-5-yl)-2,7,8,9-tetrahydro-3H-pyrido[4,3,2-de]phthalazin-3-one (BMN 673, Talazoparib), a Novel, Highly Potent, and Orally Efficacious Poly(ADP-ribose) Polymerase-1/2 Inhibitor, as an Anticancer Agent. *J Med Chem* **2016**, *59* (1), 335-357. DOI: 10.1021/acs.jmedchem.5b01498 From NLM Medline.

(2) Volpe, P.; Eremenko-Volpe, T. Quantitative studies on cell proteins in suspension cultures. *Eur J Biochem* **1970**, *12* (1), 195-200. DOI: 10.1111/j.1432-1033.1970.tb00837.x From NLM Medline.

(3) Zhao, L.; Kroenke, C. D.; Song, J.; Piwnica-Worms, D.; Ackerman, J. J.; Neil, J. J. Intracellular water-specific MR of microbead-adherent cells: the HeLa cell intracellular water exchange lifetime. *NMR Biomed* **2008**, *21* (2), 159-164. DOI: 10.1002/nbm.1173 From NLM Medline.
